# The role of community health workers in primary healthcare in the WHO-EU region: a scoping review

**DOI:** 10.1186/s12939-023-01944-0

**Published:** 2023-07-20

**Authors:** Tijs Van Iseghem, Ilka Jacobs, Dorien Vanden Bossche, Peter Delobelle, Sara Willems, Caroline Masquillier, Peter Decat

**Affiliations:** 1 Interuniversity Centre for Health Economics Research (ICHER), Department of Public Health and Primary Care, Ghent University, Ghent, Belgium; 2 Equity Research Group, Department of Public Health and Primary Care, Ghent University, Ghent, Belgium; 3 Unit Family Medicine, Department of Public Health and Primary Care, Ghent University, Ghent, Belgium; 4 Chronic Diseases Initiative for Africa, Department of Medicine, University of Cape Town, Cape Town, South Africa; 5 MENT Research Group, Department of Public Health, Vrije Universiteit Brussel, Brussels, Belgium; 6 ‘Family Medicine and Population Health’ – FAMPOP, Faculty of Medical Sciences & ‘Centre for Family, Population and Health’, Faculty of Social sciences, University of Antwerp, Antwerp, Belgium

**Keywords:** Community health workers, Primary healthcare, WHO-EU region

## Abstract

**Background:**

Existing evidence on the role of community health workers (CHWs) in primary healthcare originates primarily from the United States, Canada and Australia, and from low- and middle-income countries. Little is known about the role of CHWs in primary healthcare in European countries. This scoping review aimed to contribute to filling this gap by providing an overview of literature reporting on the involvement of CHWs in primary healthcare in WHO-EU countries since 2001 with a focus on the role, training, recruitment and remuneration.

**Methods:**

This systematic scoping review followed the guidelines of the Preferred Reporting Items for Systematic reviews and Meta-Analyses, extension for Scoping Reviews. All published peer-reviewed literature indexed in PubMed, Web of Science, and Embase databases from Jan 2001 to Feb 2023 were reviewed for inclusion. Included studies were screened on title, abstract and full text according to predetermined eligibility criteria. Studies were included if they were conducted in the WHO-EU region and provided information regarding the role, training, recruitment or remuneration of CHWs.

**Results:**

Forty studies were included in this review, originating from eight countries. The involvement of CHWs in the WHO-EU regions was usually project-based, except in the United Kingdom. A substantial amount of literature with variability in the terminology used to describe CHWs, the areas of involvement, recruitment, training, and remuneration strategies was found. The included studies reported a trend towards recruitment from within the communities with some form of training and payment of CHWs. A salient finding was the social embeddedness of CHWs in the communities they served. Their roles can be classified into one or a combination of the following: educational; navigational and supportive.

**Conclusion:**

Future research projects involving CHWs should detail their involvement and elaborate on CHWs’ role, training and recruitment procedures. In addition, further research on CHW programmes in the WHO-EU region is necessary to prepare for their integration into the broader national health systems.

**Supplementary Information:**

The online version contains supplementary material available at 10.1186/s12939-023-01944-0.

## Introduction

The Alma-Ata declaration of 1978 explicitly established health as a human right within the global health agenda, and stressed primary healthcare (PHC) as a critical mechanism for achieving Health for All [1]. Since Alma-Ata, community health worker (CHW) programmes (worldwide) have been promoted to boost health efforts within community settings [2]. CHWs who are often members of the community they serve, possess a unique understanding of the local context, including barriers and facilitators to access PHC. The American Public Health Association (APHA) defines CHWs as:

*“… a frontline public health worker who is a trusted member of and/or has an unusually close understanding of the community served. This trusting relationship enables the worker to serve as a liaison/link/intermediary between health/social services and the community to facilitate access to services and improve the quality and cultural competence of service delivery.”* [3].

This definition is one of several available [4]. CHW roles are often conceptualised as performing a bridging function and acting as connectors between professional health services and communities [5]. Evidence that CHWs can facilitate effective linkages to care can especially be found for individuals living in low-income or rural communities whose access to healthcare may be limited [2]. With the increasing burden of disease due to chronic conditions globally, PHC providers face an additional workload [6] which CHWs can help address as part of a PHC team [7]. During the COVID-19 pandemic, CHWs have become more recognised due to the need for more health workers on the ground [8–10]. CHWs can hence be relevant for global health as they help to achieve universal health coverage by delivering vital services to vulnerable and underserved populations [11] or by relieving pressure on PHC providers through task shifting [12].

CHW programmes exist mainly in Low-and Middle-Income Countries (LMICs), but are also implemented in many High-Income Countries (HICs) [13]. In LMICs, CHW engagement has resulted in major improvements in health priority areas, including reducing childhood undernutrition [14], improving maternal and child health [15, 16], expanding access to family planning services [17], and contributing to the control of HIV, malaria [18], and tuberculosis infections [13]. In many Middle-Income Countries, such as Brazil and India, CHWs are key members of the health team and are essential for providing PHC and health promotion [13, 19, 20]. In HICs, including the United States (US), Canada and Australia, evidence indicates that CHWs can contribute to reducing the disease burden by participating in the management of hypertension, cardiovascular risk factors, diabetes control, HIV infection, and cancer screening, particularly with populations living in socio-economically vulnerable circumstances [13, 21–24]. The United Kingdom (UK) has created a CHW position, *Health Trainers*, in 2004 within the National Health Service (NHS) to address health inequalities in the most disadvantaged and marginalised communities [25]. Recent systematic and scoping reviews focused on CHWs in the US, Canada, Australia or LMICs [13, 21, 26–28]. However, current literature lacks an overview of the research on CHW involvement in PHC and the role(s) they perform in the European context. Because of this, the following research question was formulated: “What is the role and what are the characteristics of CHWs involved in PHC in the WHO-EU region?”

The role, recruitment, training & remuneration of CHWs in particular are of interest because of the need to understand the mechanisms of change that lead to improved health outcomes [29]. Previous literature has pointed to the importance of CHW recruitment, training [30] and remuneration [31] in attracting, retaining and motivating CHWs. These aspects have also been included in the WHO guidelines on health policy and system support to optimize CHW programmes [32]. Accurate knowledge on the CHW’s role, recruitment, training & remuneration is of critical importance to health planners and policy makers worldwide when planning and designing (future) CHW-based interventions. This paper contributes to filling the gap in literature by describing a scoping review focusing on European CHWs, which can guide future research on CHWs and be used to inform health planners and policy makers as a key strategy in health promotion and prevention as well as a means to achieve universal health coverage in the WHO-EU region.

## Methods

A scoping review method was applied to generate an overview of the literature concerning the involvement of CHWs in PHC in the WHO-EU region since 2001. Following the research question, the PCC criteria (Population, Concept and Context) [33] were used to build the search strings [see Table 1].

**Table 1:** PCC criteria

Population = Community Health Workers	Synonyms of community health workers used across Europe: auxiliary health worker; barefoot doctor; community health practitioner; health auxiliaries; community health aide; community health officer; community health volunteer; medical auxiliary; lay health worker; village health worker [14].
Concept = Primary healthcare	This review made use of the definition of primary healthcare included in the Alma-Ata Declaration: *“Primary health care is the first level of contact for individuals and the community with the national health system and addresses the main health problems in the community, providing health promotion, preventive, curative and rehabilitative services accordingly”. Primary healthcare is not to be confused with primary care. Primary care is one aspect of primary healthcare and occurs when a trained healthcare provider diagnoses or treats a patient, usually in a clinic or hospital, at the point of entry into the health system* [1].
Context = WHO European region	The WHO-EU region was chosen as the geographical area for this review, including the following countries: Albania, Andorra, Armenia, Austria, Azerbaijan, Belarus, Belgium, Bosnia and Herzegovina, Bulgaria, Croatia, Cyprus, Czech Republic, Denmark, Estonia, Finland, France, Georgia, Germany, Greece, Hungary, Iceland, Ireland, Israel, Italy, Kazakhstan, Kyrgyzstan, Latvia, Lithuania, Luxembourg, Malta, Monaco, Montenegro, Netherlands, Norway, Poland, Portugal, Republic of Moldova, Romania, Russian Federation, San Marino, Serbia, Slovakia, Slovenia, Spain, Sweden, Switzerland, Tajikistan, The former Yugoslav Republic of Macedonia, Turkey, Turkmenistan, Ukraine, United Kingdom and Uzbekistan.

The guidelines of the *Preferred Reporting Items for Systematic reviews and Meta-Analyses, extension for Scoping Reviews* (PRISMA-ScR) [34] were used to structure the results. The review protocol can be found on Open Science Framework.

### Eligibility criteria

Table 2 displays the inclusion and exclusion criteria that were applied to guide the search. Studies written in any other language than English were translated using DeepL Translator (DeepL SE, Cologne, Germany).

**Table 2:** Inclusion and exclusion criteria

	Inclusion	Exclusion
Population	CHWs are part of the intervention or program	Study does not mention CHWs (or synonyms)
Concept	Intervention or program takes place in PHC (e.g. management of chronic diseases, cancer screenings, etc.)	Intervention or program is not related to PHC (e.g. specialised (cancer) care)
Context	Study needs to be conducted in the WHO-EU region [see Table 1]	Study takes place outside the WHO-EU region
Design	All study designs (quantitative, qualitative and mixed methods)	Not applicable
Year	Published after 2001	Published before 2001
Research question	/	Study does not provide information on at least one of the following elements: CHW role, recruitment, training or remuneration.

### Information sources

The following databases were searched for peer-reviewed literature: PubMed, Web of Science Core Collection and Embase.

### Search strategy

The PRESS checklist was used to develop search strings based on the PCC criteria mentioned above [35]. Search strings used in the different databases are presented in Additional File 1. In PubMed, Boolean operators (AND/OR), truncation and the appropriate Mesh terms were used to specify the search string. In Web of Science, the concept of ‘PHC’ was left out to broaden the search because of limited hits, but the studies were screened manually for this concept. The original search strings were launched in the databases on the 8th of February 2022. The search strategy was updated on the 9th of February 2023.

### Conducting the search and selection of the studies

**Fig. 1:**
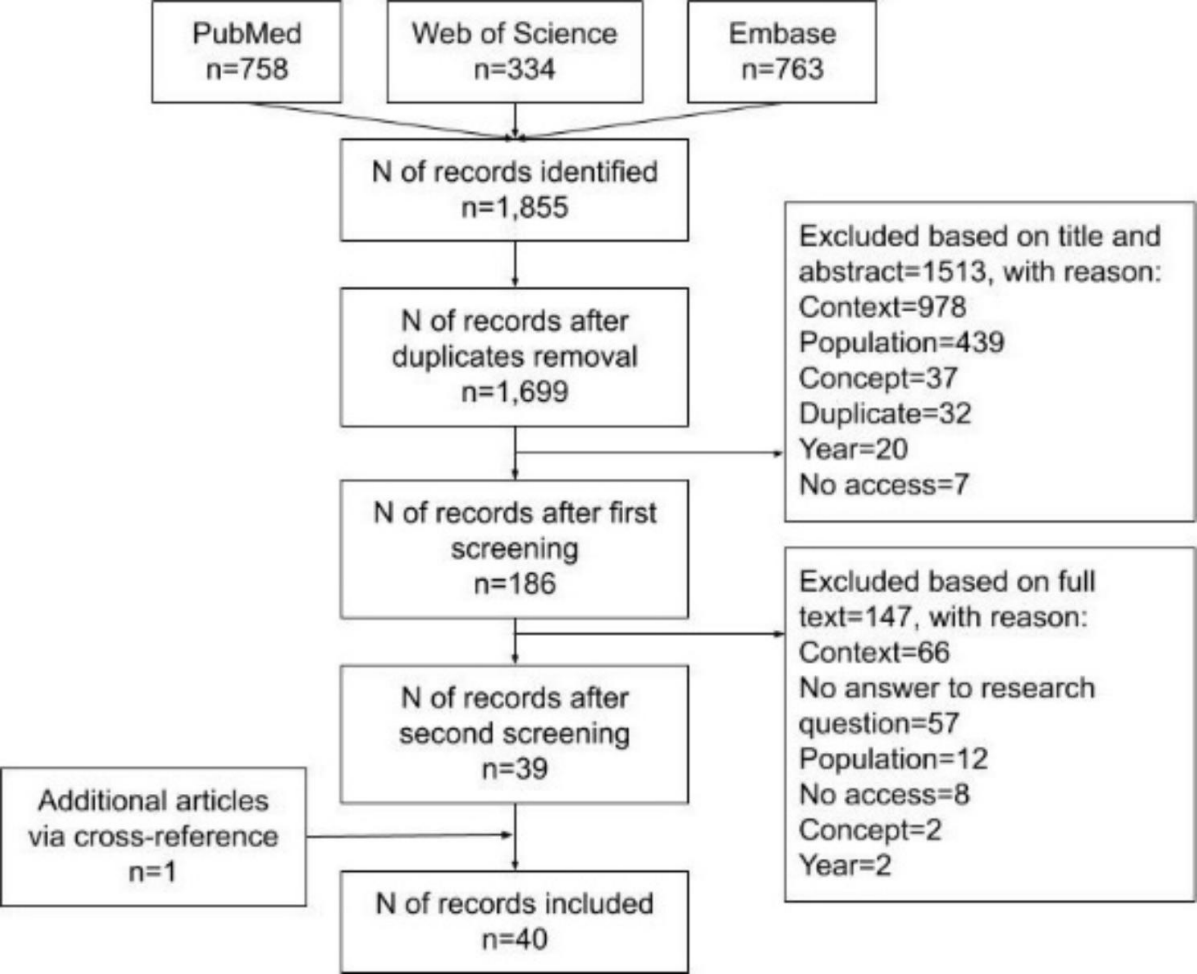
PRISMA flow chart

The resulting studies of each database were collated in Endnote Online. Duplicate studies were removed and two screening phases were carried out, including a first screening on title and abstract and a second screening on full-text reading of the studies that passed the first screening. In both screening phases the predetermined in- and exclusion criteria were assessed. Two researchers (TVI & IJ) performed the screening phases and independently screened all selected studies. Discrepancies were discussed until a consensus was found. Reasons for exclusion during both screening phases were registered and are shown in the PRISMA flowchart [see Fig. 1]. In addition, a hand search was performed to identify additional literature through backward citation tracking of included studies. These additional studies were added manually to Endnote and underwent the same screening process.

### Data extraction & data items

After screening, the selected studies were transferred to an Excel template for data extraction. The following data items were extracted: first author; year of publication; country; term used to describe CHWs; target population; area of involvement; role of CHWs; tasks of CHWs; recruitment of CHWs; training of CHWs; remuneration of CHWs; primary aim of the study; conclusions concerning CHWs; and other remarks.

### Quality assessment of selected studies

A quality assessment of the included studies was conducted to support the findings and specifically to provide more background information when interpreting the conclusions regarding the CHWs. Selected studies were subjected to the “innovative tools for quality assessment: integrated quality criteria for review of multiple study designs (ICROMS)”. This tool unifies, integrates and refines current quality criteria for many study designs [36]. A final quality appraisal was given (poor, moderate or strong) based on the minimum scores per design provided by the ICROMS tool [36]. Studies that scored lower than the ICROMS minimum score received a low-quality appraisal, scores equal to or slightly higher (+ 3) than the minimum score received a moderate quality appraisal, and a high-quality appraisal was given to scores at least four points above the minimum. Study designs for which there were no predefined criteria within the ICROMS tool (i.e., realist evaluation and costing study) were assessed on applicable criteria determined by the authors and given an appraisal based on how much their score deviated from their maximum attainable score [see Additional File 3]. Studies were not removed based on their quality.

### Data processing

The data gathered during data extraction [see Additional File 2] was summarised in a narrative form and was further analysed in the results and discussion sections below. Statistical data of the included studies was not further analysed as this was beyond the scope of this review.

## Results

### Study selection

The combined search of selected databases resulted in 1,855 studies. After the removal of duplicates, 1,699 studies remained. During the first screening on title and abstract, 1,513 were excluded, largely because they did not focus on any of the included countries. In the second screening of the full text, 147 studies were excluded, also mainly due to ineligible contexts and/or not providing the required information. Another single study was included after searching the reference lists of included studies. After both screening phases, including additional cross-references, 40 studies were included for data extraction (Fig. 1).

### Study characteristics

This review found a substantial amount (n = 40) of studies describing CHW roles, recruitment, training or remuneration in the WHO-EU. The publication year of the included studies ranged from 2005 to 2022 with 13 studies published after 2020. The included study designs showed significant heterogeneity, including: randomised controlled trials; cohort studies; quasi-experimental designs; and realist evaluations. Qualitative study designs were most prevalent among the included studies (n = 20). This scoping review identified evidence from the following countries: UK (n = 26); Belgium (n = 4); Ireland (n = 1); Hungary (n = 1); Spain (n = 2); Sweden (n = 2); Tajikistan (n = 1); The Netherlands (n = 2) and a multi-country study including twenty European countries (n = 1). Overall, 10% of studies were of low quality, 55% were of moderate quality and 35% were of high quality. The entire quality assessment table can be found in Additional File 3: ICROMS sheet & scoring system.

### Synthesis of results

Results of the individual studies were collected using a data extraction sheet which can be found in Additional File 2: Data Extraction Table. In addition, a summary table [see Table 3] and narrative synthesis of the results can be found below, structured in eight paragraphs: terminology; target population; areas of involvement within PHC; role; recruitment; training; remuneration; and evidence regarding the effect of CHW-based programmes.

**Table 3:** Summary Table of the Included Articles

First author*	Publication year	Study design	Country	Terms used for CHWs	Area of involvement	Target population
Allen-Collinson et al. [49]	2020	Qualitative study	UK	Health Trainers	Smoking cessation, improving diet, reducing alcohol intake, increasing healthy physical activity, and addressing mental wellbeing issues	‘Disadvantages’ populations in general
Ball & Nasr [50]	2011	Qualitative study	UK (Northern and central England)	Health Trainers	Healthcare access for ‘hard-to-reach’ community groups	Health trainer clients proved to be an extremely ‘hard-to-reach’, deprived group
Begh et al. (1) [57]	2011	Cluster Randomised controlled trial (RCT)	UK (Birmingham)	Outreach workers	Smoking cessation	Communities where more than 10% of the population were of Pakistani and Bangladeshi origin
Begh et al. (2) [58]	2011	Qualitative study				
Brady & Keogh [48]	2016	Qualitative study	Ireland	Traveller community health workers	Access to health services & Asthma self-management	Traveller and Roma community
Brown et al. [70]	2007	Qualitative study	UK (London and Manchester)	Lay educator	Asthma self-management	Cultural West London and inner city & socially deprived areas in Manchester
Carver et al. [61]	2012	Qualitative study	UK (Scotland)	Outreach worker	Access to care/reduce health inequalities	These workers tend to work with clients in a natural setting by visiting the populations they serve, such as homeless or drug-using populations
Cook & Wills [51]	2012	Qualitative study	UK (London)	Health Trainers	Access to health care system & health promotion	Marginalized communities, including ‘harder-to-reach and disadvantaged’ groups
Furze et al. [67]	2012	RCT	UK	Lay workers	Angina management	Adults (aged 18 + years) with a diagnosis of angina following a positive symptom-limited exercise treadmill test in rapid access chest pain clinic; does not have any exclusion criteria.
Gale & Sidhu [52]	2019	Qualitative study	UK (Midlands)	Health Trainers	Cardiovascular disease	A deprived area called the Black Country. It has a very ethnically diverse population with significant spatial segregation between ethnic groups.
Gale et al. [62]	2018	Qualitative study	UK (Birmingham)	Lay health workers & pregnancy outreach workers (POW)	Maternity care	Each locality had different characteristics of deprivation: POW#1 and POW#2 were working in an inner city community with a large migrant population, POW#3 and POW#4 were working in a suburban area of the city, adjacent to a rural area, with a predominantly white working class population and POW#5 and POW#6 were working in an inner city community, with a more established multi-ethnic community
Gilworth et al. [68]	2019	Qualitative study	UK	Lay health workers	Pulmonary rehabilitation for chronic obstructive pulmonary disease (COPD)	COPD patients
Goelen et al. [76]	2010	Individual level RCT	Belgium	Community peer volunteers	Breast cancer screening	Setting: Four semirural communities in Belgium. Sample: Women aged 50–69 years who had not had a mammogram
Hesselink & Harting [45]	2011	Qualitative study	The Netherlands	Community health workers	Maternal, newborn and child health (MNCH)	Ethnic Turkish women
Hodgins et al. [72]	2018	Quasi experimental study	UK (Scotland)	Dental Health Support Workers	Dental/oral Health	All newborn children in Scotland that are referred to a dental health support worker
Hoens et al. [39]	2021	Realist evaluation	Belgium (Brussels)	Community health workers	Provide culturally competent care	Migrant families living in deprived urban areas of Brussels
Kennedy et al. [73]	2005	Qualitative study	UK	Expert Patients Programme Trainers	Management of chronic conditions, patient education	People with chronic conditions
Kennedy, L. [69].	2010	Qualitative study	UK	Lay food and Health workers	Food and health initiatives	People from less-affluent neighbourhoods
Kenyon et al. [59]	2016	Prospective, pragmatic, individually RCT.	UK (Midlands)	POW	Maternity care	Nulliparous women under 28 weeks gestation with social risk factors
Kósa et al. [74]	2020	Quantitative analysis	Hungary	Health Mediators	Access to primary care services	Roma minority groups
López-Sánchez et al. [47]	2021	Quantitative analysis	Spain (Valencia)	Community health workers	Health literacy in the community and access to care	Persons in vulnerable situations in the city of Valencia
Lorente et al. [40]	2021	Qualitative study	20 European countries	Community health workers	Sexual health support	Men Who Have Sex with Men
Martró et al. [46]	2022	Cross-sectional study	Spain	Community Health Worker	Hepatitis care	Pakistani adults
McWilliams et al. [64]	2018	Qualitative study	UK	Lay health workers	Cancer care	5 separate lay groups: (1) completed cancer treatment; (2) friends/family of cancer patients; (3) cancer hospital volunteers; (4) cancer charity volunteers; and (5) members of the public
Netherwood [55]	2007	Pilot project	UK	Health Trainers	Access to care/ Reducing health inequalities	These areas also tend to have higher than average levels of unemployment, more single parent families and a higher proportion of black and minority ethnic groups, especially Pakistani, Bangladeshi and Caribbean communities
Rämgård & Avery [75]	2022	Qualitative study	Sweden	Lay health promotor	Health equity through health promotion	Low-income neighbourhood in the outskirts of Malmö, southern Sweden.
Roberts et al. [71]	2012	Costing study	UK	Lay educators	Asthma self-management	Eligible patients were adults aged 18 or over with clinician diagnosed asthma with persistent disease requiring regular preventative therapy. Participants also had evidence of unscheduled health care usage or increased medication for the treatment of an exacerbation in the 12 months prior to recruitment.
South et al. [63]	2012	Qualitative study	UK	Lay health workers	Health and well-being, breastfeeding, physical activity	A single community located in a disadvantaged urban area
Stone et al. [60]	2020	Qualitative study	UK	Telephone outreach workers	Cardiovascular risk assessment and management (= NHS health checks)	Black, Asian and minority ethnic (BAME) communities
Thompson et al. [56]	2018	Pilot study for RCT	UK	Health Trainers	Provide support for lifestyle change, enhance mental well-being and signpost to appropriate services	People with experience of the criminal justice system. If they have served a custodial sentence, then they have to have been released for at least 2 months. The supervision period must have at least 7 months left at recruitment.
Vanden Bossche et al. (1) [37]	2021	RCT	Belgium (Ghent)	Community health workers	Psychosocial support	Eligible patients (1) had a limited social network; (2) were older than 18 years; (3) had a psychiatric history, or a precarious social context, or an uncertain residence status, or a chronic illness, or were going through a recent critical event such as bereavement or divorce, or were older than 65 years; (4) had a score of ≤ 7 on the screening questions for emotional support and ≥ 7 on the screening questions for anxiety
Vanden Bossche et al. (2) [38]	2022	Qualitative study				CHW provided support at home to vulnerable people at risk of becoming victims of fear and social isolation during the COVID-19 pandemic
Verhagen et al. [41]	2013	Quasi experimental study	The Netherlands	Community health workers	Access to health care system	Elderly immigrants, aged 55 years and over, Living independently (alone or with others), Born in Turkey, Morocco, Moluccan Islands or descendant of Moluccan immigrants born in the Netherlands and lived in one of the Moluccan “camps”
Visram et al. [53]	2015	Qualitative study	UK	Lay health trainers	Cardiovascular risk assessment and management (= NHS health checks)	People aged 40–74 years without established disease living in socio-economic deprivation
White et al. [65]	2019	Qualitative study	UK	Lay health workers	Pulmonary rehabilitation (PR) for COPD	Persons with a diagnosis of COPD; eligibility for PR treatment; and fluency in English
White et al. [54]	2013	Mixed methods	UK	Health trainers	Chronic disease management, mental health	Areas of deprivation
Wildman & Wildman[42]	2021	Cohort study	UK (Primary practices in North East England)	Community health workers	Type 2 diabetes care	UK patients aged 40 to 74 years with type 2 diabetes in a socio-economically deprived area
Wrede et al. [43]	2021	Cohort study	Sweden	Community health workers	Migrants’ mental health status	Migrants, primarily asylum seekers and newly arrived immigrants
Yoeli & Catan [66]	2017	Qualitative study	UK	Lay public health workers	Access to health care system	Anonymised urban estate in North East England, with a long-standing reputation for its socioeconomic deprivation and poor health, yet also for its strong community spirit and friendly people.
Yorick et al. [44]	2021	Cohort study	Tajikistan	Community health workers	Maternal, newborn and child health (MNCH)	Rural farming communities in Tajikistan

*Authors are alphabetically ordered

#### Terms used to describe CHWs

Diverse terminology was used to describe the CHWs in the included studies. CHW as a term was used in twelve studies [37–48], and UK studies mainly referred to CHWs as *(lay) health trainers* [49–56]. Other studies referred *to outreach workers* [57–62], *lay (public) (health) workers* [62–69], *lay educators* [70, 71], *dental health support worker* [72], *expert patient programme trainers* [73], *health mediators* [74], *lay health promotor* [75] and *community peer volunteers* [76]. However, for the sake of readability, this paper will continue to use CHWs as an umbrella term that encompasses all these terms.

#### Target populations

The interventions in the included studies targeted diverse populations: ‘hard-to-reach’, disadvantaged, underserved, deprived, or low-income areas or groups [39, 47, 49–53, 55, 60–63, 65, 66, 69, 70, 75]; Bangladeshi and Pakistani men [46, 57, 58]; Roma groups [48, 74]; (elderly) immigrants [41, 43, 45]; people with chronic conditions [73]; nulliparous pregnant women [59]; people living with diagnosed asthma or chronic obstructive pulmonary disease (COPD) [65, 68, 71]; people living with type 2 diabetes mellitus (T2DM) in socioeconomically deprived areas [42]; new-born children [72]; angina patients [67]; psychosocial vulnerable people [37, 38]; men who have sex with men [40]; rural (farming) communities [44, 76]; and people with experience in the criminal justice system [56].

#### Areas of CHW involvement

Areas of CHW involvement within PHC showed substantial variability, broadly covering the four following categories:

1. *Access to PHC*, including: healthcare access for underserved community groups in the UK [47, 50, 51, 55, 61, 75]; guiding Roma minority groups towards PHC services in Hungary [74]; CHWs improving care for elderly immigrants in the Netherlands [41]; and enabling dialogue with health professionals for people living with asthma in Ireland [48]. Improved access was facilitated through direct referral [51] or health literacy interventions by CHWs [47]. A realist evaluation showed how training of CHWs contributed to increased access to care for migrant families in deprived urban areas [39].
2. *Management of non-communicable diseases* [54, 73], including: cancer screening [64, 76]; T2DM care [42]; angina management [67]; COPD management [65, 68]; asthma self-management [70, 71]; and cardiovascular risk assessment and management [52, 53, 56, 60]. Three studies focused primarily on smoking cessation [49, 57, 58].
3. *Psychosocial support* of migrants with regards to their mental health [43], psychosocial support in disadvantaged urban areas [63] or in the context of the COVID-19 pandemic [37, 38].
4. *Sexual and reproductive health*: maternal, new-born and child health (MNCH) [44, 45]; sexual health support for men who have sex with men [40]; and maternity care [59, 62].

Other studies focused on food and health initiatives [69], hepatitis care [46] and dental health [72].

#### Recruitment of CHWs

Information on how CHWs were recruited was lacking in nineteen studies. The remaining 21 studies reported a trend towards locally-recruited CHWs; i.e., CHWs were recruited from within the communities [54, 66, 75]. For example, Gale & Sidhu [52] recruited health trainers from the local community because they would have greater contextual and nuanced knowledge of socio-cultural barriers within the community. Similarly, Kósa et al. [74] recruited locals through advertisements at participating general practitioners’ offices. Stone et al. [60] and Verhagen et al. [41] added a slight nuance by using a recruitment strategy under supervision by a local public health commissioner or coordinator. López-Sánchez et al. [47] worked with persons that were proposed by local associations. In the study by Kennedy et al. [73], CHWs were recruited after participation in a course, implying they were also patients with chronic conditions. The studies by Brown et al. [70] and Roberts et al. [71] reported that the CHWs involved in asthma care had to have asthma themselves or at least have a relative with asthma. Brown et al. [70] reported there were no other (educational) requirements. Hoens et al. [39] recruited ten jobseekers with migration backgrounds, also without mentioning further specifications. In the study of Vanden Bossche et al. [37], CHWs needed to be aware of the problems of people living in a vulnerable context, either through experience or background. Contrary to this, Wrede et al. [43] mentioned they were looking for CHWs with a migrant background who did have some form of healthcare education. Only White et al. [65] and Yorick et al. [44] explicitly stated inclusion criteria for recruitment, including specific skills, such as networking and communications skills.

Twelve studies included information on the sex of the CHWs [39, 40, 47, 51, 57, 58, 60, 65, 66, 68, 70, 73]. Three studies reported more male CHWs: out of 20 volunteers accepted for training, eleven were male [65, 68]; 67.9% of CHWs were men [40]; four and five male CHWs participated in the focus groups [57, 58]. Eight studies reported more female CHWs: all participating CHWs were female [66]: nine out of ten CHWs were female [60]; 164 female and 37 male CHWs [47]; 15 female and four male CHWs [73]; eight female and two male CHWs [39]; all but one were female [51]; twelve female and three male CHWs [70].

#### Training of CHWs

Training of CHWs was not mentioned in five studies [42, 53, 55, 72, 76], while eleven studies stated that CHWs received training but without elaborating on its content [40, 43, 48–50, 62, 63, 66, 68, 69, 73]. In the remaining 24 studies, the reported training received ranged from two days [71] up to nine months [39]. The studies by Begh et al. [57, 58] reported two weeks of training by accredited NHS trainers and the research team. CHWs in the study of Brown et al. [70] underwent a two-day residential training, followed by a six-week distance learning programme. A twelve-week training course for CHWs was provided in the study reported by López-Sánchez et al. [47]. Kósa et al. [74] reported that CHWs were trained on-the-job with several short courses during work hours. In case of bigger project funds, longer training periods were set up. Hoens et al. [39] for example, reported a nine-month training programme for CHW supported by a European social fund with courses on culturally competent care and learning of the Dutch language, followed by an internship. Yoeli & Catan [66] concluded that training could help to engage the most underserved groups. In general, CHWs received training in delivering behavioural support, medication management, general health promotion, empowering strategies and culturally specific norms. The studies of Stone et al. [60] and Verhagen et al. [41] concluded that both purposeful recruitment and training of CHWs were vital. However, this review did not find any studies linking the duration of training with the duration of deployment of CHWs in the communities.

#### Remuneration of CHWs

CHWs received a salary in thirteen studies [41, 44, 45, 48, 52, 54, 57, 58, 62, 67, 70, 74, 75]. Five studies worked with volunteers [37, 38, 63, 65, 76] and six studies reported a mix of paid and unpaid CHWs, often without specifying the reason for this [40, 51, 65, 66, 69, 73]. In the UK-based studies, paid CHWs were financed by the NHS [52, 66] and unpaid CHWs worked for non-profit organisations [51]. White et al. reported that the volunteer CHWs were offered payment for the research elements of their role [65]. Cook & Wills [51] noted that CHWs working voluntarily offered greater potential for engaging communities and providing practical options for health gains because of their informal status compared to CHWs employed by the NHS. Remuneration was not mentioned in the other 16 studies.

#### CHW role

The role of CHWs as reported in the included studies can be classified into one or a combination of the following: educational role; navigational role; and support role.

A primarily educational role was seen in the studies of Brown et al. [70], Furze et al. [67], Kennedy et al. [73], Kennedy [69], Roberts et al. [71] and Wrede et al. [43]. CHWs in Brown et al. [70] and Roberts et al. [71] had to provide consultations and follow-up meetings to improve people’s asthma self-management. CHWs in the study of Kennedy et al. [73] were responsible for weekly educational sessions on the management of chronic conditions and Kennedy [69] reported that CHWs were mainly tasked with nutrition education. In the study by Wrede et al. [43], CHWs led the mental health sessions for Swedish immigrants. The CHWs in the study of Gale et al. [62] described themselves as *‘myth-busters’*.

Navigational roles were reported in the study of Ball & Nasr [50], focusing on underserved community groups. In the study of Begh et al. [58] in Pakistani and Bangladeshi communities in the UK, a navigational role was adopted at first, followed by a more educational role in the second stage of the intervention. Cook & Wills [51] also instructed CHWs to promote health services, next to providing some educational aspects. Kósa et al. [74] used CHWs to bridge the gap between the Roma community and the general practitioners. CHWs in Stone et al. [60] and Visram et al. [53] sent invitations to attend NHS appointments and referred to lifestyle services. Verhagen et al. [41] focused on culturally competent care and showed that this can improve access to the healthcare system. People living with T2DM were referred by CHWs to PHC practitioners in Wildman & Wildman [42] and CHWs in the study of Yorick et al. [44] referred children with malnutrition and diarrhoea to health facilities in Tajikistan. People living with COPD in Gilworth et al. [68] were assisted into pulmonary rehabilitation courses by CHWs who acted as patient navigators. Similarly, CHWs in the study of Goelen et al. [76] contacted eligible women to participate in breast cancer screenings.

A supporting role was reported in the study of Allen-Collinson et al. [49] where CHWs supported the community in making healthy lifestyle choices. Similarly, in the study of Vanden Bossche et al. [37], CHWs provided psychosocial support for patients from vulnerable communities to reduce the workload of PHC providers. In the study of Gale & Sidhu [52] and Thompson et al. [56], CHWs supported lifestyle, smoking cessation and weight management. Kenyon et al. [59] noted that CHWs supported mothers with newborn babies by regular home visits and referral to specialist services in the case of the presence of risk factors. Sexual health support was the main objective of CHWs in Lorente et al. [40]. In McWilliams et al. [64], support was provided to patients with cancer care. CHWs in Carver et al. [61] provided one-on-one support to patients before and after their health checks. Finally, CHWs in Gale et al. [62] provided informational and emotional support to pregnant women.

Finally, five included studies elaborated on the importance of the social embeddedness of CHWs in the communities they served, meaning the CHWs provided social support and were trusted points of contact. For example, Gale et al. [62] calls this *‘synthetic social support’*. Ball & Nasr [50] reported that CHWs being a ‘*person next door’* with a one-on-one approach was a critical factor in the success of their programme. CHWs viewed themselves as facilitators rather than directors and felt this was an important factor in the success of their role [50]. CHWs were also seen as friends and neighbours who are there to help the community [50]. The qualitative study by Gale & Sidhu [52] offered a nuanced explanation for intervention success in engaging communities by identifying three steps. First, CHWs should be *critical insiders*, meaning that they understand the (negative) effects of lifestyle behaviour in the community, e.g., in terms of nutrition. Secondly, CHWs should try to make small but sustainable changes to the community’s lifestyle. Third, CHWs should try to become accessible *role models* [52]. The study by Kennedy [69] identified CHWs as ‘*culturally acceptable vehicles for change*’ and highlighted that CHWs offered an alternative to sole professional interventions. South et al. [63] also concluded that social relationships are core to understanding CHW programmes.

#### Evidence regarding the effect of CHW-based programmes

Ten studies stated that CHW-based programmes proved feasible and acceptable, without reporting on the effects of the interventions [46, 53, 57, 58, 61, 64, 70, 74, 76]. Six of the included studies reported positive effects of CHW-based programmes [37, 42–44, 59, 67], including significant improvements in self-rated psychosocial health [37], less depressive symptoms in pregnant women [59], improved HbA1c levels [42], positive changes in mental health status [43], reduced anxiety and depression in people with angina [67] and improved knowledge, attitudes and practices that result in better nutrition [44]. Contrary, White et al. [54] reported they found no evidence that CHWs impacted health inequalities. Only one study mentioned the costs of CHW programmes: Roberts et al. [71] reported a lack of significant differences in the cost of training and healthcare delivery between nurses and CHWs in the UK. The generalisability of these effects could be higher given the variety of interventions across countries and the observed quality of the studies [see Additional File 3].

## Discussion

This scoping review provides the first overview of CHW involvement in PHC in the WHO-EU region and can be used to learn from past efforts, identify knowledge gaps and develop new research questions regarding the involvement of CHWs in the WHO-EU region.

The involvement of CHWs in the WHO-EU region was found in published literature spanning the last few decades, with 13 out of 40 studies published since 2020, indicating a growing interest for CHW-based programmes in European health care systems. CHW involvement was usually project-based - except in the UK - and the role, recruitment, training and remuneration of CHWs varied from context to context. The information gathered in this scoping review originated mainly from studies of moderate quality. The main explanation for this can be found in poor descriptions of managing bias in the outcome and reporting of the included studies [see Additional File 3]. In line with O’Brien et al. [77], this review recommends more consistent reporting of future research on CHW roles, recruitment, training, remuneration and other elements [78] (accreditation, equipment, supervision, and community involvement among others) of the CHW Assessment and Improvement Matrix (AIM) [78] to allow to better interpret CHW-based programme findings.

This review showed that in line with existing literature [5, 79–81], CHWs and the interventions they are engaged in are best seen as bridging the communities and the national or local health system. Within this *bridging* element the roles and characteristics of the CHWs have been adapted to the local context. This review indicates that the role of CHWs is often a combination of educational, navigational and supporting aspects, in line with the findings of a scoping review on CHW support for T2DM self-management in South Africa which found education, support and advocacy to constitute their main roles [82]. The most important CHW aspect seemed to be the social embeddedness through which trustful relationships between CHWs and their clients are created. A recent realist evaluation reported this relationship to be rooted in recognition, equality and reciprocity [38]. In line with the literature from high-income countries [83], this review found that CHWs in the WHO-EU region commonly provide services related to non-communicable diseases. Because of this, the authors of this review believe that CHWs can be an added value to reach the objectives within the Action Plan for the Prevention and Control of NCDs in the WHO-EU Region [84], making this a political priority in the region. However, CHWs are not included in the Action Plan and to our knowledge WHO-EU has not yet released any official statements regarding CHWs in the region. They did already publish some exemplary articles on CHW involvement in Albania, Azerbaijan, Turkey and Ukraine [85]. Presumably, CHWs could have a larger scope in these poorer countries and shift their focus towards disadvantaged populations in the richer countries of the region. Previous studies have identified structural and systematic barriers to access for low socioeconomic groups, such as costs, time pressure, and linguistic and cultural differences [86]. This review supports the existing evidence that CHWs can help improve access to PHC by circumventing these barriers.

Two tensions concerning the role of CHWs were also addressed in Hodgins et al. [87]: the lay vs. professional aspect of CHWs; and the CHW as service provider or as service promotor. In LMICs, the study by Hodgins et al. [87] reported that, over time, a tendency to add new functions to the CHWs’ scope of practice was reported, resulting in deprioritising certain activities, in particular promotion services. Even if governments and programme designers intend CHWs to focus primarily on health education or health promotion, communities tend to value clinical services more [87]. Therefore, CHWs tend to prioritize what their beneficiaries value most.

Recruitment strategies of CHWs were only described in more recent studies, which could be related to the increasing attention to implementation research in recent years. Most studies generally reported recruitment strategies that embody ‘insider knowledge’ [66]. This is similar to what has been described as ‘indigenous knowledge’ of Brazilian CHWs to overcome contextual challenges [88]. Locally recruited CHWs possess embodied knowledge of their communities by being part of the community. This intuitive aspect is also captured in the WHO definition of CHWs [89]. Obviously, people coming from within the community need less training than ‘outsiders’ or ‘incomers’ with limited community knowledge. For example, participating in the *Expert Patient Programme* was the only condition for recruitment in the study of Kennedy et al. [73]. Besides training-related aspects, being an locally recruited is also important when building trustworthy relationships with the community.

CHW training differed among studies and aimed to help CHWs gain skills in activities directly related to their role. This scoping review showed that the amount and type of training required should be considered in view of the local healthcare system, CHWs’ prior capacities, and the roles that CHWs are intended to take on. Training CHWs is an essential part of CHW programmes [90], but there is no consensus in the literature regarding the extent or form of the training, whether in preparation for or on-the-job training. In LMICs, training increased CHW motivation, job satisfaction, and performance, but there was no direct evidence that different aspects of training or different training approaches affected CHW performance [79]. Across health occupations there has been an evolution towards higher educational requirements and longer training periods [90]. As the number of highly trained healthcare staff grows, it is projected that this professionalisation will also affect CHWs, leaving fewer and less tasks to be performed by CHWs or requiring more professionalisation of CHWs themselves. The question arises as to how the bridging role of CHWs can be maintained. On the other hand, CHWs can relieve pressure of overburdened healthcare providers through task-shifting [12].

CHWs’ remuneration is strongly linked to their accreditation and national recognition. A US study suggested that equitable compensation for their services is an important step towards CHWs’ integration within the broader health system of the country [91]. However, compensating CHWs for certain tasks also raises the question of where the CHW role ends and another health career begins, which needs to be discussed with a view to *task-shifting* [92]. Paid work can push CHWs towards tasks they are paid for, and White et al. (2019) in their paper opined that the community basis and the cooperative nature of CHW interventions could be undermined if CHWs are remunerated. On the other hand, non-monetary incentives such as trust, respect and recognition can play an equally important role in the motivation and performance of CHWs. Nevertheless, in LMICs, the relevance of (monetary) incentives is of great importance in the planning of CHW programmes [93, 94]. The more recent studies included in this review align with the 2018 WHO guideline that CHWs should receive a financial package corresponding to their job demands, complexity, number of hours worked, training, and the roles they undertake, supported by a written agreement [31]. There is also a global push towards CHW remuneration, led by the Community Health Impact Coalition [95], and it could be valuable to compare the effectiveness of non-monetary and monetary incentives for CHWs in a European context.

This review only included articles originating from eight countries, reaffirming the initial point that there is limited evidence in the WHO-EU context and more research is needed. As a consequence of their national CHW program, 26 studies included in this review were UK-based. The NHS set up job descriptions, competencies, and an accreditation system for CHWs. However, the implementation varied across the nation and CHWs have not been recognised as a coherent occupational group [96]. Consequently, retention of CHWs has been problematic due to low pay, job insecurity, job intensity and lack of recognition within the health system. Consequently, many CHWs have moved from the NHS to non-governmental organisations [52]. Future CHW programmes can learn from the UK experience and learn from its successes and failures, knowing that integrating CHWs within the existing health care systems is a complex matter and cannot be done in silos. Health strategies (involving CHWs) must also be integrated into broader programmes focussing on poverty reduction and sustainable development [97], with a long-term vision and sustainable funding [98].

In addition, European CHW programmes can learn from lessons of CHW programmes in LMICs through reverse or reciprocal innovation [99], as shown by a pilot project in North Wales which tried to implement and learn from Brazil’s CHW strategy [100]. In 2018, WHO also published evidence-based global guidelines for health policy and systems support to optimise CHW programmes worldwide, based however on low and very low certainty of evidence [32]. Key considerations for implementation included the need to define the role of CHWs in relation to other health workers and to plan for the entire health workforce rather than specific occupational groups; to appropriately integrate CHW programmes into the existing health system; and to ensure internal coherence and consistency across different policies and programmes affecting CHWs [32].

### Limitations

The lack of a unified terminology posed difficulties for this scoping study, and nomenclature remains a fundamental challenge for studies aiming to comprehensively review CHWs programmes [80]. This review only included published studies and did not include grey literature, potentially giving rise to a publication bias. This explains why programs such as in Belgium [101] and Westminster [102] are not part of this review. A remark can be made regarding the paucity of coverage of COVID-19-related CHW-based programmes in this review. One possible explanation could be that many of these programmes are ongoing and/or the corresponding studies are yet to be published in scientific journals. This review was also limited to a few aspects of importance to CHW programmes as described in the WHO guideline for CHW programmes [32] and CHW AIM framework [78]. Finally, although literature published in a language other than English was translated into English, this did not rule out a language bias because of the English search strategy. In spite of the growing interest in CHWs stated before, this review did not find literature for 45 countries in the WHO-EU region. This is possibly a consequence of the combined language and publication bias.

## Conclusion

This scoping review indicated that CHWs provide a wide range of health-related services in the WHO-EU region, albeit in a limited number of countries. This review found substantial variability in recruitment, training and remuneration. In general, most studies reported a trend in favour of locally recruited CHWs, with some form of training and payment in most of the included studies. Their roles were classified into one or a combination of the following: educational, navigational and supporting roles. The most important aspect of CHW-based programmes was the social embeddedness in the communities they served. Further research on CHW programmes in the WHO-EU region is necessary to prepare for their integration into broader national health systems.

### Recommendations

Based on the topics addressed in this review, some recommendations can be made to inform future research and policymaking. First, future research projects involving CHWs should mention their involvement and elaborate on the role, training and recruitment of CHWs to obtain a full picture of the programme. In addition, other elements of the CHW AIM framework [78] (accreditation, equipment, supervision, and community involvement among others) need to be taken into account to enable evaluation of CHW involvement. Second, there is a need for a more rigid evaluation of the evidence stated in this scoping review. Systematic reviews or realist syntheses on the role of CHWs in the WHO-EU region can respond to this research need. Third, there is limited high-quality evidence regarding CHWs’ ability to improve access to PHC for marginalised, vulnerable and underserved populations in the WHO-EU region, especially when compared to the amount of evidence from other high-income countries, such as the US. This indicates the need for rigorous studies and program evaluations. Finally, the cost and cost-effectiveness of CHW interventions, CHW involvement and integration in PHC settings in the WHO-EU region is still unknown, pointing to the need for health economics analysis (or similar). At policy level, it might be time to move from project-based CHW-based programmes to building meaningful long-term partnerships between CHWs, communities and policy-makers backed by sustainable funding [28, 98]. Therefore, national governments and the health sector should clearly commit to CHW-based programmes. Governments in the WHO-EU region should better recognize and support sustainable CHW-based programmes [40] as e.g. shown in the UK.

## Electronic supplementary material

Below is the link to the electronic supplementary material.

Additional File 1: Search Strings per database

Additional File 2: Data Extraction Table

Additional File 3: ICROMS sheet & scoring system

## Data Availability

The dataset(s) supporting the conclusions of this review is(are) included within the article (and its additional file(s)).

## List of Abbreviations

AIM: Assessment and Improvement Matrix
APHA: American Public Health Association
CHW: Community Health Workers
HICs: High-Income Countries
ICROMS: Integrated Quality Criteria for Review of Multiple Study Designs
LMICs: Low- and Middle-Income Countries
NHS: National Health Service
PRISMA: Preferred Reporting Items for Systematic reviews and Meta-Analyses
PRISMA-ScR: Preferred Reporting Items for Systematic reviews and Meta-Analyses, extension for Scoping Reviews
UK: United Kingdom
US: United States
WHO: World Health Organisation
WHO-EU: WHO European Office
