# Supplementary material for: The role of community health workers in primary healthcare in the WHO-EU region: a scoping review
Source: Int J Equity Health. 2023 Jul 20;22:134. doi: 10.1186/s12939-023-01944-0 (PMC10357780; doi:10.1186/s12939-023-01944-0)
Supplement: Supplementary file 1 — Additional File 1: Search Strings per database [file 12939_2023_1944_MOESM1_ESM.pdf]

Data source	Search string	Number of records 08/02/2022 (records from 2001-2021)	Update 09/02/2023 (records from 2022-09/02/2023)	Total number of records at 09/02/2023
PubMed (filter 2001-2021)	("Community Health Workers"[Mesh] OR "Community Health Work*" OR "auxiliary health work*" OR "barefoot doctor*" OR "community health aide*" OR "health auxiliar*" OR "health educator*" OR "health promotor*" OR "community health aide*" OR "community health officer*" OR "community health practitioner*" OR "community health volunteer*" OR "community health work*" OR "medical auxiliar*" OR "lay health work*" OR "outreach work*" OR "village health work*") AND<br>("Primary Health Care"[Mesh] OR "Primary care" OR Primary health care OR primary care practice OR primary care OR first line care)<br>AND<br>("Europe"[Mesh] OR Europ* OR EU OR Albania OR “Albania”[Mesh] OR Andorra OR “Andorra”[Mesh] OR Armenia OR “Armenia”[Mesh] OR Austria OR “Austria”[Mesh] OR Azerbaijan OR “Azerbaijan”[Mesh] OR Belarus OR “Republic of Belarus”[Mesh] OR Belgium OR “Belgium”[Mesh] OR Bosnia and Herzegovina OR "Bosnia and Herzegovina"[Mesh] OR Bulgaria OR “Bulgaria”[Mesh] OR Croatia OR “Croatia”[Mesh] OR Cyprus OR “Cyprus”[Mesh] OR Czech Republic OR "Czech Republic"[Mesh] OR Denmark OR “Denmark”[Mesh] OR Estonia OR “Estonia”[Mesh] OR Finland OR “Finland”[Mesh] OR France OR	673	85	758

	"France"[Mesh] OR Georgia OR "Georgia"[Mesh] OR Germany OR "Germany"[Mesh] OR Greece OR "Greece"[Mesh] OR Hungary OR "Hungary"[Mesh] OR Iceland OR "Iceland"[Mesh] OR Ireland OR "Ireland"[Mesh] OR Israel OR "Israel"[Mesh]OR Italy OR "Italy"[Mesh] OR Kazakhstan OR "Kazakhstan"[Mesh] OR Kyrgyzstan OR "Kyrgyzstan"[Mesh] OR Latvia OR "Latvia"[Mesh] OR Lithuania OR "Lithuania"[Mesh] OR Luxemburg OR "Luxembourg"[Mesh] OR Malta OR "Malta"[Mesh] OR Moldova OR "Moldova"[Mesh] OR Monaco OR "Monaco"[Mesh] OR Montenegro OR "Montenegro"[Mesh] OR Netherlands OR "Netherlands"[Mesh] OR Republic of North Macedonia OR "Republic of North Macedonia"[Mesh] OR Norway OR "Norway"[Mesh] OR Poland OR "Poland"[Mesh] OR Portugal OR "Portugal"[Mesh] OR Romania OR "Romania"[Mesh] OR Russia OR Russian Federation OR "Russia"[Mesh] OR San Marino OR "San Marino"[Mesh] OR Serbia OR "Serbia"[Mesh] OR Slovakia OR "Slovakia"[Mesh] OR Slovenia OR "Slovenia"[Mesh] OR Spain OR "Spain"[Mesh] OR Sweden OR "Sweden"[Mesh] OR Switzerland OR "Switzerland"[Mesh] OR Tajikistan OR "Tajikistan"[Mesh] OR Turkey OR "Turkey"[Mesh] OR Turkmenistan OR "Turkmenistan"[Mesh] OR Ukraine OR "Ukraine"[Mesh] OR United Kingdom OR "United Kingdom"[Mesh] OR UK OR Uzbekistan OR "Uzbekistan"[Mesh])			
Web of Science (Search on Topic, All	TS=((”Community Health Worker*" OR "auxiliary health worker*" OR "barefoot doctor*" OR "community health aide*" OR "health auxiliaries*" OR "health educator*" OR "health promotor*" OR	313	21	334

databases, 2001-2021)	“community health aide*” OR “community health officer*” OR “community health practitioner*” OR “community health volunteer*” OR “community health worker*” OR “medical auxiliar*” OR “lay health worker*” OR “outreach worker*” OR “village health worker*”)<br>AND<br>(Europe OR EU OR Albania OR Andorra OR Armenia OR Austria OR Azerbaijan OR Belarus OR Belgium OR “Bosnia and Herzegovina” OR Bulgaria OR Croatia OR Cyprus OR “Czech Republic” OR Denmark OR Estonia OR Finland OR France OR Georgia OR Germany OR Greece OR Hungary OR Iceland OR Ireland OR Italy OR Kazakhstan OR Latvia OR Lithuania OR Luxemburg OR Malta OR Moldova OR Monaco OR Montenegro OR Netherlands OR “Republic of North Macedonia” OR Norway OR Poland OR Portugal OR Romania OR Russia OR “San Marino” OR Serbia OR Slovakia OR Slovenia OR Spain OR Sweden OR Switzerland OR Turkey OR Ukraine OR “United Kingdom” OR UK OR Israel OR Kyrgyzstan OR Tajikistan OR Turkmenistan OR Uzbekistan))			
Embase (Advanced search (“Map to preferred term in Emtree”, “Search also as free text in	('community health worker'/exp OR 'community health worker' OR 'auxiliary health worker*' OR 'barefoot doctor*' OR 'health auxiliaries*' OR 'health educator*' OR 'health promotor*' OR 'community health aide*' OR 'community health officer*' OR 'community health practitioner*' OR 'community health volunteer*' OR 'community health worker*' OR 'medical auxiliar*' OR 'lay health worker*' OR 'outreach worker*' OR 'village health worker*')<br>AND	621	142	763

all fields", "Explode using narrower Emtree terms", and "Search as broadly as possible", 2001-2021)	('europe'/exp OR europe OR eu OR 'albania'/exp OR albania OR 'andorra'/exp OR andorra OR 'armenia'/exp OR armenia OR 'austria'/exp OR austria OR 'azerbaijan'/exp OR azerbaijan OR 'belarus'/exp OR belarus OR 'belgium'/exp OR belgium OR 'bosnia and herzegovina'/exp OR 'bosnia and herzegovina' OR 'bulgaria'/exp OR bulgaria OR 'croatia'/exp OR croatia OR 'cyprus'/exp OR cyprus OR 'czech republic'/exp OR 'czech republic' OR 'denmark'/exp OR denmark OR 'estonia'/exp OR estonia OR 'finland'/exp OR finland OR 'france'/exp OR france OR 'georgia'/exp OR georgia OR 'germany'/exp OR germany OR 'greece'/exp OR greece OR 'hungary'/exp OR hungary OR 'iceland'/exp OR iceland OR 'ireland'/exp OR ireland OR 'italy'/exp OR italy OR 'kazakhstan'/exp OR kazakhstan OR 'latvia'/exp OR latvia OR 'lithuania'/exp OR lithuania OR 'luxemburg'/exp OR luxemburg OR 'malta'/exp OR malta OR 'moldova'/exp OR moldova OR 'monaco'/exp OR monaco OR 'montenegro'/exp OR montenegro OR 'netherlands'/exp OR netherlands OR 'republic of north macedonia'/exp OR 'republic of north macedonia' OR 'norway'/exp OR norway OR 'poland'/exp OR poland OR 'portugal'/exp OR portugal OR 'romania'/exp OR romania OR 'russia'/exp OR russia OR 'san marino'/exp OR 'san marino' OR 'serbia'/exp OR serbia OR 'slovakia'/exp OR slovakia OR 'slovenia'/exp OR slovenia OR 'spain'/exp OR spain OR 'sweden'/exp OR sweden OR 'switzerland'/exp OR switzerland OR 'turkey'/exp OR turkey OR 'ukraine'/exp OR ukraine OR 'united kingdom'/exp OR 'united kingdom' OR 'uk'/exp OR uk			

	OR israel OR ‘israel’/exp OR kyrgystan OR ‘kyrgystan’/exp OR tajikistan OR ‘tajikistan’/exp OR turkmenistan OR ‘turmenistan’/exp OR uzbekistan OR ‘uzbekistan’/exp)<br>AND<br>'primary health care'			
Total number of records		1,607	248	1,855
Number of records after removal of duplicates		1,482	217	1,699
