# Supplementary material for: The role of community health workers in primary healthcare in the WHO-EU region: a scoping review
Source: Int J Equity Health. 2023 Jul 20;22:134. doi: 10.1186/s12939-023-01944-0 (PMC10357780; doi:10.1186/s12939-023-01944-0)
Supplement: Supplementary file 2 — Additional File 2: Data Extraction Table [file 12939_2023_1944_MOESM2_ESM.pdf]

Data extraction table

#	First author	Publication year	Study design	Country	Term used for CHWs	Area of involvement	Role of the CHWs + relevant information on this role	Tasks of the CHWs	How were the CHWs recruited?	Were the CHWs trained?	Were the CHWs paid?	Aim of the study	Conclusion(s) regarding CHWs	Target population	Remarks + sex or gender of the CHWs
[49]	Allen-Collinson et al.	2020	Qualitative study	UK	Health Trainers	Smoking cessation, improving diet, reducing alcohol intake, increasing healthy physical activity, and addressing mental wellbeing issues	Promote healthy living and help people make healthier lifestyle choices and, ambitiously, to reduce health inequalities in local communities by supporting clients in developing and sustaining healthier lifestyles	Translating knowledge and supporting clients in smoking cessation, reducing alcohol intake, increasing exercise and physical activity levels, and having healthier diets + referring, or signposting their clients to other specialist health services and professionals for issues deemed beyond their jurisdiction.	Not mentioned	Yes, no further information	Not mentioned	The researchers sought participants' experiences and perceptions of the HT role, and how they both individually and collectively made sense of, and constructed meanings around their occupational identity	Such sustained support work, whilst not accorded high status within the healthcare hierarchy generally, nevertheless may be vital in translating esoteric, scientific, bio-medical knowledge into public and community understanding, in order to achieve lasting healthy-behaviour change amongst those most at risk of, and from, unhealthy behaviour and health problems, particularly in	disadvantages populations in general	Sociological theory + not mentioned

												disadvantaged communities.		
[50]	Ball & Nasr	2011	Qualitative study	UK (Northern and central England)	Health Trainers	healhcare access for 'hard-to-reach' community groups	CHW as a 'person next door' working to improve the health of their own communities. Health trainers were unanimous in feeling that their role was not clearly defined and, in practice, was not quite what they had expected. They emphasized a number of key challenges facing this new role at an operational level. The first was the 'real tension' between pressures for the health trainer programmes to have a national (standardized) profile versus the need to maintain a 'local' focus. Health trainer initiatives must endeavour to remain flexible and responsive to their own neighbourhood needs, rather than workers and services being constrained into one 'stereotypical' role. Heath trainers viewed themselves primarily as facilitators rather than directors and felt that this was an extremely important factor in the success of the role. Not being perceived as a health professional but being viewed instead as 'still human and having their weaknesses' also made health trainers more	the provision of such support, in conjunction with psychological skills to alter behaviour, can help to reduce inequalities in health	Not mentioned	Yes, no further information	Not mentioned	To examine the perceived value of the health trainer scheme.	A critical factor in this success appears to be the unique combination of time, the 'person next door' and a 'one-to-one' approach, which facilitated an innovative and highly productive connection between the health trainer and client. However, participants in this evaluation perceived that the current format and constituents of service performance data were significantly failing to credit the health trainer scheme with the full extent of this impact.	Health trainer clients proved to be an extremely 'hard-to-reach', deprived group	Study from the early days of the UK National Health Trainer Programme. Need for cost-effectiveness studies. + not mentioned

							approachable and closer to their potential clients. Indeed, health trainers felt that people living in deprived areas viewed them as ‘friends and neighbours who are there to help them'.							
[57]	Begh et al. (1) Begh et al. (2)	2011	Cluster randomized controlled trial	UK (Birmingham)	Outreach workers	Smoking cessation	These outreach workers come from the ethnic groups of interest (i.e. two of Bangladeshi origin and two of Pakistani origin) and live in the communities that they serve. Between them, they speak several relevant languages (i.e. Sylheti, Bengali, Punjabi, Mirpuri, Urdu and English).	During the first phase, i.e. November 2007 to May 2008, outreach workers concentrated on referring people to existing services that included pharmacies, drop-in clinics, and general practices. The second phase ran for six months from June 2008 to November 2008 and concentrated on outreach workers combining more limited outreach with providing treatment for smokers directly, rather than always referring to NHS services.	Not mentioned	Outreach workers had two weeks of training in delivering behavioural support and medication management for smoking cessation, general health promotion, communication skills, and the cultural specific norms of Pakistani and Bangladeshi smokers. The training involved role-	Yes, fixed salary	To pilot a cluster randomised controlled trial comparing the effectiveness of Pakistani and Bangladeshi smoking cessation outreach workers with standard care to improve access to and the success of English smoking cessation services	The intervention proved feasible and acceptable. Outreach workers expanded reach of smoking cessation services in diverse locations of relevance to Pakistani and Bangladeshi communities. Given these data and the data on the increasing use of services by Pakistani and Bangladeshi smokers nationally, it is clear that outreach might have a role to play, but the mainstay of reaching these smokers is attracting	Bangladeshi and Pakistani men, communities where more than 10% of the population were of Pakistani and Bangladeshi origin	4 and 5 male CHWs participated in the focus groups
[58]			Qualitative study	UK (Birmingham)	Outreach workers	Smoking cessation	Although outreach workers successfully expanded service reach, they faced the challenges of perceived lack of awareness of the health risks associated with smoking in older smokers and apathy in younger smokers. These were compounded by perceptions of "lip service" being given to their role by community organisations and tensions both amongst the outreach workers and with the wider management team.								

										playing the activities in outreach in English and in minority languages. All outreach workers were assessed as competent based on these role-plays by the end of training. The training was delivered by accredited NHS trainers and the research team.			them to general NHS services and the NHS has been successful at doing so over the past year.		
[37]	Vanden Bossche et al. (1) Vanden Bossche et al . (2)	2021	Randomized controlled trial	Belgium (Ghent)	Community health workers	psychosocial support	The promising role for CHWs as a strategy to reach out to vulnerable communities, to identify problems early, and to support patient follow-up indicates potential to reduce unnecessary workload burden on primary care professionals. Our findings support the potential of CHW	They take on the following tasks: to detect problems and to inform and advise, support, stimulate, and empower vulnerable patients.	The CHWs are volunteers who, from their background or experience, are more	Training + supervision	Volunteers	This randomized controlled trial (RCT) tested whether a primary healthcare (PHC) based community health worker (CHW)	We failed to find a significant effect of the intervention on the prespecified psychosocial health measures. However, the intervention	Eligible patients (1) had a limited social network; (2) were older than 18	An important condition for implementation in PHC practice is that CHWs are

							interventions as a task shifting strategy to reduce family physicians’ and other primary care professionals’ workload.		aware of the problems of people in a vulnerable context.			intervention could tackle psychosocial suffering due to physical distancing measures in patients with limited social networks.	did lead to significant improvement in self-rated change in psychosocial health. This study confirms partially the existing evidence on the effectiveness of CHW interventions as a strategy to address mental health in PHC in a COVID context	years; (3) had a psychiatric history, or a precarious social context, or an uncertain residence status, or a chronic illness, or were going through a recent critical event such as bereavement or divorce, or were older than 65 years; (4) had a score of ≤7 on the screening questio	integrated in primary care teams. Future research should therefore take a closer look at this organizational embeddedness of CHW strategies in the primary care team. If this further research demonstrates the benefits we postulate, then there would be a good case for scaling up this approach in a HIC setting.

														ns for emotional support and ≥7 on the screening questions for anxiety	+ not mentioned
[38]		2022	Qualitative study				They can serve as role models for clients. The role of CHWs to offer psychosocial support to vulnerable people with limited social networks and suffering from loneliness and anxiety was sometimes mentioned to be demanding.	CHWs provided hands-on, tailored support to clients spanning the domains of social support, coaching, advocacy, and navigation to healthcare if needed.	Volunteers from the community	All CHWs were trained using 2 online training modules of 2 h, entailing communication skills, providing correct information, recognizing alarming signals presented by clients, and safety measures to prevent COVID-19 infection. Additional on-demand support		This study aimed to unravel mechanisms and contextual factors that determine the trust in a CHW program offering psychosocial support to vulnerable citizens during the COVID-19 pandemic.	CHWs are a crucial public health outreach strategy for PCP and complement and enhance trust-building by primary care professionals. In the process of building trustful relationships between CHWs and clients, different mechanisms and contextual factors play a role in the trustful relationship between primary care professionals and patients.	CHW provided support at home to vulnerable people at risk of becoming victims of fear and social isolation during the COVID-19 pandemic	/ + not menioned

										was provided, and peer-to-peer coaching was provided in small groups once a month.					
[70]	Brown et al.	2007	Qualitative study	UK (London and Manchester)	Lay educator	Asthma self-management	There was also a perception of the role being important.<br>Lots of uncertainty in their role, especially at the beginning. This may in part have reflected the high level of nurse turnover, which occurred within the practices throughout the study. It emerged that working in a busy practice as a temporary outsider, with very little interaction with the permanent staff can be lonely. This sense of isolation was lessened when the lay educator was treated as staff and made to feel at home. Having patients who benefited from their skills enforced the perception of their role being worthwhile, thereby enhancing satisfaction. There is a need to address the lay educators' feelings of frustration and self reproach at patients not attending appointments within their training and at follow up mentoring sessions. It also emerged that the NRTC trainers'	Nurses and lay educators were asked to offer an initial consultation of up to 45 min for each patient, and to follow this with a second face-to-face reinforcing session of up to 30 min duration, three weeks after the first consultation. The lay educators' involvement with each patient was approximately 13 months (from initial face-to-face visit; face-to-face follow-up 3 weeks later then three monthly telephone follow-up for 12 months). A standard written asthma action plan template (adapted from one provided by the national asthma charity Asthma UK and in use in clinical practice at the London site) was provided for all	Recruited by advertisement. Criteria for selection were that the trainer or a close relative had to have asthma. No minimum educational qualifications were stipulated.	Yes, the lay persons underwent a two-day residential training course at the National Respiratory Training Centre (NRTC, now part of Education for Health) followed by a 6 week distance learning programme. Overall the NRTC training was viewed as a	The lay educators were paid £8.00/h (€11.98/ $15.28), from which they paid their own tax, national insurance and travel expenses. Although the lay educators were paid a nominal sum, monetary reward was dismissed as an incentive to take part in the project;	To capture the experiences and feelings of lay educators in an asthma self-management programme to aid understanding of optimal methods of recruitment, training and retention, and to enhance their value within the programme.	Lay educators are a potential resource for giving self-management education to patients with long-term conditions such as asthma. However, there are some considerations that need to be taken into account regarding contracts, retention and continual support.	cultural West London and inner city, socially deprived areas in Manchester	Practice implications: Lay educators need a flexible but comprehensive training programme, contracts, on site mentoring and support. They seem most contented when welcomed by health professionals and treated as part of the

							and project leaders' confidence in the lay educators, helped their own self beliefs about their ability to carry out their role as a lay educator.	educators in the study and this was individualised for each patient, advising them when to increase their routine preventative therapy, when to start a course of steroid tablets, and when to seek urgent medical attention.		positive experience, alongside 'demanding' and 'exhausting' words such as 'interesting', 'excellent' and 'informative' were used to describe their experience.<br>+<br>Wish for role play. Provide a less intense training course over a longer period.	indeed few educators claimed all that was due to them.<br>+<br>Lay educators to be tied into a formalised contract. Inform lay educators during their training of basic professional conduct and this to be set out as rules in their contract.				team. + 3 male and 12 female CHWs
[51]	Cook & Wills	2012	Qualitative study	UK (London)	Health Trainers	Access to health care system & health promotion	Health trainers emphasized their similarities with their communities and underestimated their differences.<br>Most health trainers did not consider themselves to be professionals but aspired to be, or on some level already were, professionals.<br>Approaches to, and experiences of, community	Engagement with communities ranged from simply promoting services, to advocacy or to creating social networks and group activities.<br>Among the five non-PCT health trainers, the aim of community engagement, which was part of their	Not mentioned	All participants received training, however very diverse levels and systems of education.	Mix between paid (NHS) and unpaid (third sector = non profit) participants	The aim of this study was to explore the experiences and approaches adopted by health trainers in engaging with marginalized communities.	There remains a lack of clarity about the role of the health trainer. Lay workers are not necessarily part of the marginalized communities they are	Marginalized communities, including harder-to-reach and disadvantaged groups	There is a tension between assumed lay identity versus the adoption of a formalized role

							engagement varied according to their employing organization. Lack of clarity about the health trainer role persists at an operational level, including confusion about its various forms. It raises questions about the different types of lay workers, assumptions about their community membership, and their best use within the public health workforce.	existing role, was mostly to raise awareness of particular health conditions amongst at-risk groups, or to provide practical opportunities for the community to lead healthy lifestyles, such as running exercise classes					expected to engage, while their ability to do so is compromised by the professional culture of the NHS and its approach to community engagement. Health trainers based in the community or voluntary sector appear to offer greater potential for engaging communities and providing those communities with practical opportunities for health gain.		and move to a paraprofessional status. There is also a tension between a statutory objective of individual behaviour change versus the health benefits of greater community control and empowerment. + all but one were female with diverse ethnic backgrounds
[52]	Gale & Sidhu	2019	Qualitative study	UK (Midlands)	Health Trainers	Cardiovascular disease	Three important theoretical steps for understand how health promotion activities are carried out by CHWs in	Health trainers were equipped to: monitor blood pressure, take height and weight	Health trainers were recruited from the	Training was varied and often needs	The HT service was funded, free at	Make consideration of how CHWs themselves understand,	This study offers a much more nuanced explanation	A deprived area called the	It is important to note that, in

							their own communities: first, that being a critical insider enables CHWs to make sense of the diverse constraints on lifestyles within their community; second, that they understand their own role as constrained by operating within this context so that they aim to support clients to make small but sustainable changes to their lifestyles, and third, that the uncertainties and tensions of translating population based risk information to individual clients is (at least partially) resolved at an embodied level, with the CHWs becoming accessible role models for the value of changed lifestyles.	(to calculate body mass index), complete glucose testing, provide lifestyle, smoking cessation and weight management support, as well as encouraging increased physical activity.	local communities they served so they would have greater contextual and nuanced knowledge of the socio-cultural barriers faced by the population they treated. A key selection criteria was health trainers being chosen because they too had completed a significant lifestyle change and demonstrated skills of building and maintaining relations	based with many health trainers developing skills which focused more greatly on clinical aspects of their role. Health trainers were supervised by one designated manager whereby performance was monitored by meeting key performance indicators determined by the local healthcare provider. The formal training most receive	the point of access, by the National Health Service (NHS) via outsourcing to not-for-profit, community interest companies	interpret and experience their role and attempts to understand this experience within the wider social context of the new public health, within a risk society	for the intervention's successes in engaging communities in new ways with health promotion, and its limitations in terms of failing to address the underlying causes of health inequalities.	Black Country. It has a very ethnically diverse population with significant spatial segregation between ethnic groups.	line with trends more generally in the UK context to deliver community-based health Risk work or resilience work services via not-for-profit organisations or social enterprises rather than the public sector (National Health Service), health trainers had moved from being NHS employees to being employ

									hips with clients that would lead to behavioural change. The recruitment of CHWs relies on applicants bringing with them certain knowledge and skills, so that rather than needing to be trained on the wider context of (non-health) risks, soft skills in communication, local knowledge of the environment and activities available, they have this already	would be around how to apply risk assessment tools, facilitating behavioural change, or the health promotion and education messages they are expected to deliver.					ed by a not-for-profit, community interest company. The retention of CHWs remains problematic, notably due to low pay, job insecurity, the emotionally and practically intensive nature of the role, and their poorly recognised para-professional status in a professionalised health system. + not mentioned

[39]	Hoens et al.	2021	Realist evaluation	Belgium (Brussels)	Community health workers	provide culturally competent care	One participant was especially strengthened in her role as CHW through opportunities to participate actively in meetings at the home care organisation	Deliver culturally competent services for home care organisations	10 jobseekers with migration backgrounds to become CHWs in Brussels (Belgium).	A 9-month programme consisted of courses on culturally competent care and the Dutch language, an internship in a home care organisation, and formal exchange moments between CHWs and care employees. In addition, a 5-day training course on culturally competent care was organised specifically for care employees + internshi	Not mentioned. A European Social Fund supported the training, no information on remuneration of CHWs	Three research questions were formulated: (a) What increase in the cultural competence of the home care organisations can be identified at the end of the project? (b) How did the training contribute to this increase? (c) Which factors and preconditions made the positive outcomes of the training more likely?	This paper concludes that although the project contributed to a shift in organisational culture towards cultural competence, it remains challenging to effect a similar shift in the deep structure of care organisations	Migrant families living in a deprived urban areas of Brussels	Focus on training + 8 women and 2 men

										p: by being involved as interns in the home care organisations, CHWs gained extensive knowledge and developed self-confidence in their work.					
[73]	Kennedy et al.	2005	Qualitative study	UK	Expert Patients Programme (EPP) Trainers	management of chronic conditions, patient education	On the face of things, the potential of the UK EPP workforce is that it crosses the line between notions of formal and informal work, but it also raises questionsabout the nature of the role and identity which trainers will assume. However, as Williams & Sibbald (1999) noted, diversification of primary care workforce roles risks creating role uncertainty and the adoption of more than one role identity is possible. Expert Patients Pro-gramme trainers could claim a distinct ‘professional’ identity on the bases of group socialisation and the development of particular skills as part of being trainedto deliver self-	The EPP training package aims to enable participants to more effectively manage their conditionthrough the development of problem-solving skills andthe opportunity afforded by the group to exchangeideas and suggestions. The package is an anglicised ver-sion of the Chronic Disease Self-management Programdeveloped by researchers at Stanford University in the USA (Lorig et al. 1999), and consists of six, 2.5-	Recruited after participation in the course	Participation in the EPP training packages was seen as preparation.	Volunteer vs paid trainers. It is possible to see a potential career path-way with people entering either from within the healthservice or directly from the patient level, starting as avoluntar	The aim of the present paper is to assess the establishment and prospects of these trainers as a new workforce role in the EPP and the NHS.	Working on a voluntary basis would ease the administrative burden for the PCT, it would run the risk of makingit harder to integrate the EPP into existing NHS provision of health care, losing the opportunity to link in with professional practice and reaping the benefits of self-care support as	People with chronic conditions	Pros and cons of voluntary vs paid in discussion + 15 female and 4 male

							management courses. Alternatively, the CHW could continue to see their main identity as that of a lay person or ‘expert patient’.<br>"More role modelling than teaching."	hour ses-sions held on a weekly basis. The content is highlyscripted (topics covered include: relaxation, diet, exer-cise, tiredness, breaking the symptom cycle, managingpain and medicines, and communication with health-care professionals), and the groups are made up of 10 –16 people with a long-term condition (which may beself-defined) who have self-referred to the course			y tutor. There were expectations on the part of trainers thatbeing employed in the NHS would provide security and opportunities which were unavailable in the voluntary sector where pay and conditions were accepted as being inferior. However, these expectations were undermined by concerns about the long-term viabilityof their role because of the		part of the broader agenda of deliveringcare to people with long-term conditions.		

											uncertainty about the future of the programme within the NHS				
[69]	Kennedy, L..	2010	Qualitative study	UK	Lay food and Health workers (LFHW)	Food and health initiatives	The primary role for LFHWs was to encourage dietary change by translating complex messages into credible and culturally appropriate advice. Lay roles spanned three broad areas: nutrition education; health promotion; and administration and personal development.<br>Narratives from both professionals and LFHWs indicated that the primary role for LFHWs was to encourage dietary change by translating complex messages into credible and culturally appropriate advice.<br>Nonetheless, LFHWs were confident of the benefits resulting from a skill mix and believed they offered a suitable alternative to professional-only delivered services.<br>Indeed, a major theme is the role of lay people as 'culturally acceptable vehicles for change'.<br>Only minor credit was given to LFHWs for their role in benefiting the wider community.	Delivering local food and health initiatives, to improve understanding of the perceived benefits associated with their involvement and wider opportunities for promoting health	Not mentioned	Yes, no further information	Paid and unpaid	To explore the experiences of lay food and health workers (LFHW) and professionals	This study highlights previously unreported benefits related to the direct experiences of lay people used in community nutrition in the UK, which go beyond those associated with professional-led initiatives, suggesting the need to adopt a broader view of lay involvement in the UK public health workforce.	less-affluent neighbourhoods	UK health services have been actively recruiting lay people into roles previously restricted to health professionals + not mentioned

[59]	Kenyon et al.	2016	Prospective, pragmatic, individually randomised controlled trial.	UK (Midlands)	Pregnancy Outreach Workers (POWs)	Maternity care	The POW service was developed before the trial began, but not available outside the trial, and was provided by an independent organisation, who had access to supervision from experts with specific skills and knowledge.	POWs were trained to provide individual support and case management for the women including home visiting from randomisation to 6 weeks after birth. Standard maternity care (control) included provision for referring women with social risk factors to specialist midwifery services, available to both arms. POWs also provided breast feeding and advice about infant care	Not mentioned	POWs were trained to provide individual case management for the women including home visits, and were integrated into the community midwifery teams	Not mentioned	We sought evidence of effectiveness of lay support to improve maternal and child outcomes in disadvantaged families.	This trial demonstrates differences in depressive symptomatology with addition of the POW service in the powered subgroup of women with 2 or more social risk factors. Addition to existing evidence indicates benefit from lay interventions in preventing postnatal depression	Nulliparous women under 28 weeks gestation, with social risk factor	/ + not mentioned
[74]	Kósa et al.	2020	Quantitative analysis	Hungary	Health Mediators	access to primary care services	In this respect, health mediators shifted towards the role that community health workers fulfill in primary care in many countries outside of Europe.<br>They participated in the organization and operation of various preventive services as specified by the public health coordinator (their supervisor) and the GP, being specifically responsible for increasing attendance at the health status assessment.	Their major task was to bridge the gap between general practitioners and their socioeconomically vulnerable clients by ensuring individualized support for the latter, counterbalancing the potential increase of health inequalities inherent in the population approach of prevention uncovered by	Recruited from local communities with no requirement for professional or vocational training. They were recruited by public advertisement as prescribed by law	Received training on the job: a number of short courses of continuing education were also developed for health mediators and completed during work	Yes, health mediators worked under part-time employee contracts equivalent to 20 work hours per week<br>All expenses related to both vocational and mediator	The paper describes the contribution of the work of health mediators to the uptake of two new services provided by group practices.	The future of general practices lays in multidisciplinary teams in which health mediators recruited from the serviced communities can be valuable members, especially in deprived areas.	In Roma minority groups	/ + not mentioned

								Frohlich and Potvin. Health mediators were also involved in various health education activities facilitated by training, relevant material and printed leaflets for distribution. Mediators also had administrative tasks of reporting their work, including participation at the monthly meetings of the GP cluster.	and by locally distributed leaflets facilitated by the participating GPs and practice nurses. All health mediators were required to reside in the local community; preference was given to those applicants who identified with or had experience working with the largest minority (Roma) population of the regions.	hours No requirement for professional or vocational training in advance	trainings were fully paid for by the Programme, and both were completed during work hours. (The Model Programme had been implemented in the framework of the Swiss Contribution Programme SH/8/1. The project was supported by a grant from Switzerland through the Swiss Contribution.)				
[47]	López-Sánc	2021	Quantitative analysis	Spain (Vale	Community healt	Health literacy in the communi	The main role of the persons trained as CHWs in this case is to act as a bridge between	Not mentioned	persons who have been proposed	The training-action course	Not mentioned	To analyse the profile of the persons and	The results of the CHW training-action course	persons in vulnerable	/ + 164 women and 37 men

	hez et al.			ncia)	h workers	ty and access to care	associations (and their communities) and the health services, especially when their peers are experiencing a situation of inequality due to a lack of information about access to and functioning of services and programs.		by associations (immigrant, local or intercultural)	lasts for 120 hours over 12 weeks: 80 theoretical and 40 practical, given in two sessions of 4 hours every week. The theoretical part is divided into three modules, and after each one there are 2 weeks to complete the practical part.		associations that participated in the course, quantify peer education activities and analyse their evolution.	improve over time given that a significant increase in participation by associations and women can be seen, along with a greater number of activities completed during the training. One effect of this is that CHWs are contracted or carry out voluntary activities in the associations	situations in the city of Valencia	
[40]	Lorente et al.	2021	Qualitative study	20 European countries	Community health workers	Sexual health support	CHWs as a public health workforce contribute to all steps of the continuum of services for HIV, viral hepatitis, and other STIs amongst MSM in Europe. Female and volunteer CHWs are generally more common in high inequality countries, while paid and peer CHWs are more common in low inequality countries, which suggests that the socio-political	Most CHWs worked/volunteered in private not-for-proft organisations (86.4%). CHWs involvement in the continuum of services for HIV, viral hepatitis and other STIs was as follows: primary prevention (88.6%), consultation and counselling (58.0%),	Not mentioned	90% received training	71% paid, 19% volunteer	Explore the role of Community Health Workers (CHWs) who work in non-clinical settings to provide sexual health support around HIV, viral	National governments should recognise and support CHWs better in order to make their activities more visible and sustainable, and increase their impact	Men Who Have Sex with Men	Results of European Community Health Worker Online Survey (ECHOES) Only in non-

							environment limits the willingness and/or ability of MSM to perform CHWs roles and engage as peer CHWs in Eastern Europe.	testing provision (50.6%), linkage to care (49.8%), and treatment and support activities (51.3%). CHWs were also involved in cross-cutting activities such as developing interventions, advocacy, and engaging in research (46.3%).				hepatitis, and other sexually transmitted infections (STIs) to men who have sex with men (MSM) in Europe and neighbouring countries.	on the continuum of services.		clinical settings + mostly men (67.9%) .
[64]	McWilliams et al.	2018	Qualitative study	UK	Lay health workers	Cancer care	In line with literature suggesting that LHWs act as a supportive link with health care, general consensus was for the role to be devel-oped alongside general practices and embedded within community groups. This coincides with the recommendation that social networks providing education can help overcome attendance barriers forsuspected cancer symptoms, particularly in lower socioeconomic groups. There were no major differences between groups in whatthe role should look like although the post-treatment and friends/family groups felt that their experience of cancer would add "expertise."This should be interpreted with caution given the potential persuasive nature of the role; it is worthwhile to note that the	<u>Setting</u>: Each group agreed that LHWs should be community based and linked to primary care, workplaces, or local associations where connections to community members are pre-established <u>Audience</u>: shared characteristics between LHWs and the audience were viewed as increasing salience and engagement to maximise success <u>Modality</u>: LHW roles were viewed as face-to-face or telephone-based with aninformal conversation style. LHW charachteristics: A focus on personality characteristics, friendly,non-judgemental,	Not mentioned	Yes, several participants felt that training should be individually tailored. Participants felt that LHWs should have a clearlydefined remit, and any intervention should be underpinned byappropriate levels of training and	Not mentioned	The present study aimed to explore the acceptability and feasibility of LHW interventions focussing on cancer prevention/early diagnosis within the UK	LHW interventions to promote early diagnosis or screening were generally considered acceptable in a UK context. LHW interventions focussing on reducing cancer risk may be less feasible.	5 separate lay groups: (1) completed cancer treatment; (2) friends /family of cancer patients; (3) cancer hospital volunteers; (4) cancer charity volunteers; and (5) members of the public	/ + not mentioned

							groupsviewed clear role descriptions and training as essential to minimise this.	empathetic, and light-hearted but resilient, was used to describe effective LHWs.<br>Clear boundaries: all participants discussed the necessity for a well-defined LHW role with distinct boundaries to limit risk-related situations. Risk wasdiscussed in relation to liability protection for any LHW programme		support. Findings highlight the need for LHW structured training focussing on knowledge and skills requirements for effective intervention delivery. LHWs should have access to ongoing support from professionals, similar to findings from LHW in other fields					
[71]	Roberts et al.	2012	Costing study	UK	Lay educators	Asthma self-management	It is crucial that the role of the lay educator is well defined and the employment contract is well defined.<br>The employment contract was not well defined and it is difficult to perceive whether individuals were acting as an expert member of staff or volunteer. This has implications on the success	The protocol intervention for both the lay educators and nurses offered patients two face-to-face consultations plus follow up support.	Who had personal or family experience of asthma	The lay educators received a 2 day Education for Health training course with follow-up distance	Lay educators were involved in the trial on a part-time basis and were not contractually employed	A costing study was undertaken using the trial data to account for the cost of delivery of the service under both strategies and the resulting impact on	There were no significant differences in the cost of training and healthcare delivery between nurse and lay trainers, and no significant difference in the cost of	Eligible patients were adults aged 18 or over with clinician diagnosed asthma	/ + not mentioned

							of the individual at delivering the outcomes and more research needs to be carried out on the role, expectations and effectiveness of expert patients in trials and clinical practice as well as the roll-out cost implications. It would be considerably cheaper to use lay educators as a volunteers but further work on the implementation of lay educators into "real-world" clinical practice needs to be carried out to ensure this is the best option.			learning and three 1 day training sessions and on the job mentorin g.		unscheduled healthcare (measure of effectiveness) in this trial	unscheduled health care use.	with persist ent disease requiri ng regular preven tative therap y. Partici pants also had eviden ce of unsche duled health care usage or increas ed medica tion for the treatm ent of an exacer bation in the 12 mon ths prior to recruit ment.	
[63]	Sout h et al.	2012	Qualita tive study	UK	Lay healt h work ers	Health and well-being, breastfee ding, physical activity	lay health workers were perceived as adopting a caring role and actions that might seem relatively low key, such as making refreshments, were described in terms of	Volunteer lay health workers supported the delivery of varied health promotion activities. Volunteer role	Not mentione d	Voluntee r walk leaders received a short training and then	Voluntee rs	This paper examines lay interpretatio ns of lay health worker roles within three UK	The paper concludes that social relationships are core to understandin g lay health	a single commu nity located in a disadva ntaged	/ + not mention ed

							facilitating participation in health activities. Service capacity: LHWs had fewer time constraints than professionals + insight in the community: Lay health workers were seen as having both a bonding role in bringing people together in these social groups and a bridging role in connecting people to services. Barriers for LHWs were bureaucracy and literacy.	included: staffing community health events and regular activities (e.g. exercise classes); signposting to local services; peer support to service users; support to a smoking cessation service hosted by the project; health education within the community.		independ ently led communi ty group walks		community-based health promotion projects	worker programmes and therefore analysis needs to take account of the capacity for community members to move within a spectrum of participation defined by increasing responsibility for others.	urban area	
[60]	Ston e et al.	2020	Qualita tive study	UK	Tele phon e outr each work ers	cardiovas cular risk assessme nt and manage ment (=NHS health checks)	Some TOWs reported not feeling qualified to carry out signposting; however others welcomed this role, and were recognized as having better knowledge of locally available lifestyle services than PCP staff, as well as having time, skills and motivation to engage personally with the patients they called.	The outreach call included an invitation to an NHS Health Check appointment, lifestyle questions, and signposting to lifestyle services.	Under supervisi on of a Local Authority public health commissi oner, recruitm ent targeted individual s with communi ty knowledg e/connec tions, skills such as language interpret ation and patient advocacy or other relevant experien	Yes, by Local Authority public health commissi oner. Training was offered on the purpose and content of NHS Health checks, the EMIS template (electroni c patient record system used in the PCPs) used to record patient response	Not mentione d	We examined the experiences of staff delivering the intervention	To maximize the potential of telephone outreach to impact equity, purposeful recruitment and training of TOWs is vital, along with support and integration of TOWs, and the telephone outreach intervention, in participating PCPs	Focus on Black, Asian and minorit y ethnic (BAME) commu nities.	/ + nine out of ten female

									ce (e.g. alcohol addiction counselling/smoking cessation). Recruitment of the right individuals to work as TOWs was seen as important by PCP staff, who highlighted attributes such as local knowledge, strong motivation and good telephone communication skills.	s, and motivational interviewing to help resolve patients' ambivalence about behaviour change to encourage uptake of NHS health checks. Motivational interviewing training was well received by TOWs, with several participants reporting that it improved their confidence in conducting the outreach call.					
[41]	Verhagen et al.	2013	Quasi experimental study	The Netherl	Community health	Access to health care system	First of all, it is important that the immigrant elderly reached by the community health workers also include	In the first step, the community health worker conducts home visits to the elderly to examine	Community health workers are selected	Yes, the training consists of two, six hour	The community health worker will be	We developed an intervention programme in which	This study can contribute to the improvement	Elderly immigrants: Aged 55	A randomised controlled trial

				ands	workers		the more difficult-to-reach frail elderly. Additional, proper identification and recruitment of the community health workers are crucial for a successful implementation of the intervention in this study. Finally, commitment of local community based health care or social welfare organisations is needed to start up culturally sensitive care and integrate this care into their existing services.	health problems, barriers to health care and social welfare services, and needs for adequate care<br>In the second step, the community health worker identifies commonly shared problems based on the home visits and organises problem focused working groups of eight to twelve elderly persons<br>In the third step, the community health worker cooperates with the elderly and providers of health care and social welfare services in finding solutions and in creating and conducting improvement programmes.<br>In the fourth step, these new initiatives will be implemented by the local providers of health care and social welfare facilities in their existing health care and welfare services in collaboration with the elderly	from local ethnic communities (on site). A local programme coordinator will preselect community members who have the qualities and skills required to become a community health worker. Therefore, only community health workers with deep roots and strong ties within the local migrant community will be selected. Therefore, the community health	sessions. During the sessions, the trainers explain and discuss the intervention programme. The sessions have an interactive character and contain exercises to practice necessary skills and a role play with an actor. Furthermore, the research team will discuss the research process and the collaboration between the community health workers	paid as a parttime worker who is connected to, but not a staff member of one of the local social welfare organisations involved to keep their independent role as intermediary.	ethnic community health workers act as liaisons between immigrant elderly and local health care and social welfare services. In this study we evaluate the effectiveness and the implementation of this intervention programme	of care for elderly immigrants by developing culturally sensitive care whereby they actively participate. To enable a successful transition, proper identification and recruitment of community health workers is required. Taking this into account, the study aims to provide evidence for an approach to improve the care and access to care for elderly immigrants	years and over, Living independently (alone or with others), Born in Turkey, Morocco, Moluccan Islands or descendant of Moluccan immigrants born in the Netherlands and lived in one of the Moluccan “camps”	whereby the community health workers randomise participants to specific conditions is difficult to translate into practice, because this may conflict with their role as a community health advocate due to the assignment to control conditions with no direct benefit to the community members they serve + not

									workers will be identified and recruited by using a profile consisting of necessary qualities and skills.	and the research team.					mentioned
[53]	Visram et al.	2015	Qualitative study	UK	Lay health trainers	cardiovascular risk assessment and management (=NHS health checks)	The health trainers have this, kind of, unique role because of their translation of evidence and risk factors into a format that people would understand. So I think with the community programme, we will see more people going into lifestyle programmes—stop smoking, physical activity, weight management—as a result of having a health check as opposed to those people having a health check done in general practice.	This involved taking the person's weight, height [to calculate body mass index (BMI)] and blood pressure, as well as asking key screening questions. Those found to be eligible for a full health check were invited to a separate appointment, where their waist circumference and cholesterol level (via pinprick blood testing) were measured and the results were explained. The health trainers also provided brief advice and signposted to lifestyle services, where appropriate.	Not mentioned	Not mentioned, but: Health trainers and others involved in delivering health checks require appropriate training to communicate the level of risk without creating unnecessary anxiety or false reassurance.	Not mentioned	to determine the feasibility, acceptability and uptake of a community-based NHS health check service delivered by non-clinicians and to explore the likely impact of the service in terms of health improvement and a reduction in health inequalities	A community-based, health trainer-led approach may add value by offering an acceptable alternative to health checks delivered in primary care settings. The service appeared to be particularly successful in engaging men and younger age groups. However, there exists the potential for intervention-generated inequalities	people aged 40–74 years without established disease living in socio-economic deprivation	/ + not mentioned
[65]	White et al.	2019	Qualitative study	UK	Lay health	Pulmonary rehabilitation (PR)	The high regard that many successful completers of the treatment have for PR suggested that they may	Support patients, meet with patients, make a digital recording for	Inclusion criteria: diagnosis of COPD;	Recruited volunteers participat	Volunteers LHWs were	This study was designed to evaluate the feasibility	PR-experienced COPD patients can	persons with a diagno	/ + 55% male volunteers

					workers	for chronic obstructive pulmonary disease (COPD)	be good candidates for the LHW role. Our patient advisory group, comprising PR experienced COPD patients, felt that the voluntary status of the LHW was a key element. They felt that the community basis of the concept and the cooperative nature of PR itself could be undermined if the LHWs were not volunteers.	evaluation, attend meetings, inform research team, provide interviews, telephone calls, mentoring meetings…	completion of PR in the previous 18 months; .40 years; competent in English; independently mobile; able to use a smartphone; willing to undertake LHW training; willing to support up to eight newly referred COPD patients over 6 months; willing to make digital recordings of all patient contacts. Exclusion criteria: current life-threatening illness; serious	ed in a 3-day training course commissioned from the Royal Society of Public Health	offered payment for the research elements of the LHW role (recordings of interactions and a feedback interview)	of a cluster randomized controlled trial to test the efficacy of lay health workers (LHWs) in improving the uptake and completion of pulmonary rehabilitation (PR) in the treatment of COPD	be recruited, trained, and retained as LHWs to support participation in PR, and can deliver the intervention. Participant COPD patients found the intervention acceptable. A cluster randomized controlled clinical trial is feasible.	sis of COPD; eligibility for PR treatment; and fluency in English	accepted for training

									mental illness					
[42]	Wildman & Wildman	2021	Cohort study	UK (Primary practices in North East England)	Community health workers	Type 2 diabetes care	England's publicly funded National Health Service (NHS) is currently introducing a program of social prescribing, enabling primary care teams to refer patients to a link worker, which is a type of CHW who facilitates access to sources of voluntary and community sector support	Patients were referred by a primary care practitioner to a link worker (CHW), who helped patients identify condition management and social needs goals across 8 domains, covering lifestyle, self care, symptom management, work and volunteering, money, living conditions, social relationships, and mental well-being.	Not mentioned	Not mentioned	Not mentioned but funded by NHS	To determine whether a UK National Health Service (NHS) CHW social prescribing program was associated with improved hemoglobin A1c (HbA1c) levels among patients with type 2 diabetes	A social prescribing program with referral to CHWs targeting patients' social needs and health behaviors was associated with improved HbA1c levels, suggesting that holistic CHW interventions may help to reduce the public health burden of type 2 diabetes	UK patients aged 40 to 74 years with type 2 diabetes in a socio economically deprived area	/ + not mentioned
[43]	Wrede et al.	2021	Cohort study	Sweden	Community health workers	Migrants' mental health status	The role of the health communicators was to facilitate a trusting atmosphere, to function as a bridge between the participants' previous and current environment, and to adapt the health messages to the respective cohort's level of education, needs, and interests. The intent was to encourage and frame empowering discussions around set health topics rather than to mediate unidirectional information. In sum, the program can be described as the	Five 2–3 h sessions (held once a week), is described in its manual to: "strengthen a positive development of mental health through providing the individual with tools for coping with minor mental health issues". The sessions were led by community health workers	So called health communicators, who themselves had migrant backgrounds, some form of health care education (e.g., nursing, pharmacy)	Yes, additional training in group dynamics and the 'Hälsostöd' content.	Not mentioned	To investigate the association between participation in a health promotion program and changes in migrants' mental health, and if socio-demographic factors and length of time in the new home country,	We conclude that psycho-educative programs, similar to 'Hälsostöd', have potential for promoting asylum seekers' and newly arrived immigrants' mental health as the evaluation showed a considerable number of	Migrants, primarily asylum seekers and newly arrived immigrants	/ + not mentioned

							promotion of mental health through increased health literacy and 'sense of coherence', and the opportunity to, together with peers, elaborate on personal experiences					Sweden, influenced a potential association.	positive changes in participants. The result suggests the importance of offering immigrants health promotive programs in close connection with arrival to the new home country		
[66]	Yoeli & Catan	2017	Qualitative study	UK	Lay public health workers	Access to health care system	Lay health knowledge of an individualUK LPHW is determined primarily by his or herposition within or in relation to the communitywithin which he or she works. Insider LPHWs pos-sess an embodied knowledge and incomer LPHWspossess an experiential knowledge which, althoughdifferent from one another, are essentially interper-sonal in nature	Not mentioned	Insider vs incomer: services recruiting LPHWs should decide whetherthey are seeking embodied insider LPHW knowledge, experiential incomer LPHW knowledge or a mixture of both. All recruitment practices will nevertheless need	Training shouldbe regarded as a mutual and reciprocal process. Opportunities for LPHWs to pass on their knowledgeand skills to services should be inserted into existing training programmes	Some LPHWs worked as health trainers or volunteered in structured health promoting roles within local statutory and voluntary services	This study sought to discover the lay knowledge ofhealth trainers and other LPHWs, aiming to ascertain how this knowledge might more effectively be utilised within UK public health services	Lay health knowledge can take different forms,and different LPHWs can provide different forms of lay health knowledge. Public health structures and services in the UK should makebetter use of all forms of LPHW knowledge, and should seek from LPHWs training on how to engage the most 'hard-to-reach' or	anonymised urban estate in North East England, with a long-standing reputation for its socioeconomic deprivation and poor health, yet also for its strong community spirit and	Although elsewhere in the world LPHWs are expected to come from the communities within which they work and know that their knowledge is valued, neither is the case for LPHWs in the

									to remain mindful of relevant equalities legislation				'difficult-to-engage' groups.	friendly people.	UK + all participating CHWs were female
[44]	Yorick et al.	2021	Cohort study	Tajikistan	Community health workers	Maternal, newborn and child health (MNCH)	CHWs and CAWs mutually encourage health behavior change, reinforce better agricultural practices, and promote maternal and child health and nutritious diets through household visits, community events, and peer support groups. THNA advocated for a specific role and responsibilities assigned for community volunteers in the government health system, and additional resources required for HLSCs for facilitating the work of community volunteers. These resources are necessary, at a minimum, for training community volunteers and travel by HLSC staff to the communities for mentoring and supervision.	CHWs refer children with malnutrition and diarrhoea and pregnant women who are not registered for antenatal care to health facilities	Criteria included: at least secondary school education; selfmotivation and interest in community volunteer work; ability to devote at least 8 hours a week; good networking and communication skills; positive relationships with neighbors, village leaders, and health providers; not being currently	Yes, THNA provided an initial training for CHWs over 5 days on all relevant topics and trained CAWs for a total of 8 days, 2 days per quarter, on seasonal topics relevant to the upcoming agricultural season	Yes, through the Tajikistan Health and Nutrition Activity (THNA), funded by the U.S. Agency	The article describes THNA's implementation approach from 2018 to June 2020 and uses 2016 data as a baseline.	Community volunteers specializing in agriculture or MNCH and WASH are an effective workforce for improving individual knowledge, attitudes, and practices that result in better nutrition	Rural farming communities in Tajikistan are disproportionately poorer, more food insecure, and undernourished compared to other communities.	/ + not mentioned

									employed as a health worker (for CHWs); and availability to travel 1 day a month to the district center for peerlearning meetings.						
[76]	Goelen et al.	2010	Individual level randomized trial	Belgium	Community peer volunteers	Breast cancer screening	/	Community peer volunteers made up to three attempts to call the women in the intervention arm.	Not mentioned	Not mentioned	No, volunteers	To assess the effect of a tailored telephone reminder call by community peer volunteers on mammography rates in women who do not attend a breast cancer–screening program.	The tested telephone reminder call is suitable for Belgian women. The telephone reminder call may be implemented in settings similar to the studied context.	Setting: Four semirural communities in Belgium. Sample: Women aged 50–69 years who had not had a mammogram.	/ + not mentioned
[48]	Brady & Keogh	2016	Qualitative study	Ireland	Traveler community	Acces to health services & Asthma	While they are not health professionals, they are specifically trained and trusted members of the Traveller community who	their role encompasses a number of activities including identification of	Not mentioned	basic training not disclosed additiona	employed by the Health Services Executive	The aim of this evaluation was to determine	The train-the-trainer approach was shown to be an effective,	Traveller and Roma community	/ + not mentioned

					health workers	self-management	have access to the communities	health care needs, developing and disseminating health information and materials, advocacy at individual and community level and enabling dialogue with health professionals + astha education		l asthma education programme	on a part-time basis for up to 12 hours per week	the cultural and educational appropriateness of a pilot asthma education programme developed for the Traveller and Roma community in Ireland.	transferable and cost effective strategy to building capacity to outreach specialist asthma education to a wider population. There was a very positive response to the programme among the stakeholders, facilitators and the participants generally.		
[61]	Carver et al.	2012	Qualitative study	UK (Scotland)	outreach worker	acces to care/reduce health inequalities	two distinct roles: to provide one-to-one support to patients who have had a health check and are thought to require additional support; and to form a link between practices and local voluntary organizations. The role was perceived as vague with little agreement about what the role involves, particularly at the beginning of the project.	provide support and signposting to Keep Well patients following their health check. The OW will meet with the patient, determine their needs and the support available to meet these needs, and then signpost or refer patients to services such as counselling or smoking cessation.	Not mentioned	Keep Well outreach workers are trained to use behavioural change techniques, based on motivational interviewing,4 to support patients to change their lifestyle	Not mentioned	The aim of this study was to understand how staff and patients view the Keep Well outreach worker role.	The outreach worker role was viewed positively, particularly in terms of partnership working with practices and local services, and the benefits of support to patients.	These workers tend to work with clients in a natural setting by visiting the populations they serve, such as homeless or drug-using	Although there appears to be a consensus that OWs work with hard-to-reach, disadvantaged or underserved populations. + not mentioned

										and improve their health.				populations	
[62]	Gale et al.	2018	Qualitative study	UK (Birmingham)	lay health workers & pregnancy outreach workers	Maternity care	synthetic social support. These LHWs provided instrumental, informational, emotional and appraisal support to the women they worked with, which are all key components of social support. POW#4 described their role as 'myth-busters'.	POWs provided education/information to the women, such as entitlements to free milk, child tax credits, drug or alcohol use and specific training about weaning, healthy eating or parent craft.	The POWs came from a range of backgrounds and has various career routes prior to applying for this role: all except one were women; they were from a range of ethnic backgrounds, they ranged in age from 20s to 40s. Not mentioned how.	trained, but not professionally qualified	They were paid modestly but above the living wage in the UK	To understand the nature and context of the POWs' everyday work	The 'promise' of lay health workers to deliver improvements in health outcomes and reduction in health inequalities at low cost may be unrealistic, but that does not mean that they are without value in the system.	Each locality had different characteristics of deprivation: POW#1 and POW#2 were working in an inner city community with a large migrant population, POW#3 and POW#4 were working in a suburban area of the city, adjacent to a rural area, with a predo	/ + not mentioned

														minantly white working class population and POW#5 and POW#6 were working in an inner city community, with a more established multi-ethnic community	
[68]	Gilworth et al.	2019	Qualitative study	UK	Lay health workers	pulmonary rehabilitation for chronic obstructive pulmonary disease (COPD)	To assist COPD patients newly referred to Pulmonary Rehabilitation in attending the course and act as a patient navigator	to support up to eight patients referred to pulmonary rehabilitation over a period of up to twelve months, to attend three training sessions, to support the patient, to speak with, meet and accompany the patient	In the feasibility study, LHWs were recruited from COPD patients who had previously completed PR	Yes, Some LHWs may have needed more time in training, with greater attention to repetition of role play exercises	voluntary, the intervention lasted one year	The aim of this qualitative evaluation was to investigate the experiences of COPD patients referred to PR and supported by trained, PR-experienced volunteer LHWs.	The common bond between LHWs and patients of having COPD together with the LHWs positive, first-hand experience of PR were dominant and recurring themes in their relationship.	COPD patients	Poor rates of PR uptake and PR completion. + out of 20 volunteeers accepted for training, 11 were male
[45]	Hesselink &	2011	Qualitative study	The Ne	Community	Maternal, newborn	CHWs were treated as an integrated part of midwifery care and had to	The CHWs were involved in additional tasks,	CHW had tohave a good	The CHWs had	The CHWs were	This article is a report of an evaluation of	A culturally sensitive perinatal	ethnic Turkish women	The community

	Harting			therlands	health workers	and child health (MNCH)	adapt perinatal programmes to reach minorities.	such as interpreting during midwife consultations, helping midwivesduring home visits, and giving pregnant women informationon specific topics during extra consultations	command of the Dutch language	alreadyreceived training in doing individual consultations and giving group classes on health- and pregnancy-related issues forminorities. They received an additional 2-day training duringwhich they received instruction on how to accurately implement the programme in accordance with the pro-gramme handbook.	contracted for a number of hours per week.	a multiple risk factor perinatalprogramme tailored to ethnic Turkish women in the Netherlands.	programme is able to gain access to a hard-to-reach minority group at increased risk for poor perinatal health outcomes.Such a programme may be well received and potentially effective		health workers' Turkish background was vital in overcoming cultural and language barriers and creating a confidential atmosphere + not mentioned
[54]	White et al.	2013	Mixed methods	UK	Health trainers	chronic disease management,	the aim of reducing inequalities in health by providing 'support from next door rather than advice from on high' to	Specific for each project	Health trainers were intended to be	Not specified	No volunteers although two	An analysis of eight evaluations was undertaken	No evidence was found that health trainers were impacting on	areas of deprivation	In all the evaluations, the services

						mental health	people living in those communities who wanted to make lifestyle changes		recruited from disadvantaged communities		services worked with local volunteers for outreach work, thus freeing up health trainers for one-on-one work	and findings compared to national monitoring data collected through the DCRS to assess how local findings compared to the national picture.	health inequalities at a population level		that were analysed aimed to address health inequalities either through being based in disadvantaged areas (as measured by deprivation scores linked to super output areas (SOAs), receiving referrals from professionals working in those areas and/or doing targeted outreach work + not mentioned

[72]	Hodgins et al.	2018	Quasi experimental study	UK (Scotland)	Dental Health Support Workers	Dental/oral Health	The role of the DHSW is to link these families with a dental practice, promote oral health behaviour change, and link families to wider community resources. Once a family is referred by a health visitor, a DHSW will make contact when the child is around 3 months of age	Not specified: "The content of the DHSW intervention should be tailored to the family's needs."	Not mentioned	Not mentioned	Not mentioned	This study evaluates the effectiveness of Dental Health Support Workers (DHSW) at linking targeted families with young children to primary care dental practices.	Link workers (DHSW) within the Childsmile programme are effective at linking targeted children to primary care dental services and, most notably, at a younger age for prevention	All newborn children in Scotland that are refered to a dental health support worker	This work is part of the wider evaluation of the Childsmile programme. + not mentioned
[46]	Martró et al.	2022	Cross-sectional study	Spain	Community Health Worker	Hepatitis care	education, screening and simplified access to treatment	Urdu-speaking community health agents assisted participants in completing it when required.	Not mentioned	These agents were trained in rapid HCV testing, DBS collection and pre- and post-test counselling.	Not mentioned	The aims of the HepClink study were (i) to implement and assess the quality of a micro-elimination strategy based on a community intervention and (ii) to obtain data from primary care (PC) registries as a baseline comparator	This novel community intervention was well accepted and effective at reaching a Pakistani migrant population with a low-level knowledge of HCV	The community intervention targeted Pakistani adults	In this study, decentralization and task-shifting for both complete HCV screening (performed by Pakistani community health agents) and treatment assessment and delivery were feasible and

														effective at facilitating access to diagnosis + not mentioned
[75]	Rämgård & Avery	2022	Qualitative study	Sweden	Lay health promotor	Health equity through health promotion	The function of local “brokers” working as LHPs was thus outlined in the initial funding application	Deliver health promoting interventions using a multisectoral approach, which would take into consideration local needs, through actively involving communities, in an attempt to reduce health inequity	The LHPs were recruited in the neighbourhood during large citizen workshops. But first, they need to be accepted as individuals by the residents they are serving.	the LHPs were in the course of the programme trained in PAR methodologies and Freire's dialogues about empowering communities.	Employed part-time	The purpose of this article is to describe the role of the lay health promoters in the initiative and discuss the challenges of their position mediating between residents of the neighbourhood and the established institutions	Programmes needs to offer sufficient resources and freedom of action to engage in activities that actually benefit the community, in line with the priorities expressed by the inhabitants.	Low-income neighbourhood in the outskirts of Malmö, southern Sweden.	/ + not mentioned
[67]	Furze et al.	2012	Randomized controlled trial	UK	Lay workers	Angina management	facilitate a lay angina management programme, under the supervision of community cardiac rehabilitation nurses.	It includes a workbook and a relaxation programme on CD, and was introduced in a 45-minute interview by a lay facilitator during which misconceptions about living with angina were dispelled, and goals to increase physical activity and reduce	The facilitators were six lay people with experience of heart disease, either personally (myocardial	The lay workers were four women and two men who were trained face-to-face, in 40 hours over 4 weeks with	The cost of a lay worker per hour was £6,15 whilst the travel costs per home visit was assumed at £5 per trip.	To compare a lay-facilitated angina management programme with routine care from an angina nurse specialist.	The angina management programme produced some superior benefits when compared to advice from a specialist nurse.	Adults (aged 18+ years) with a diagnosis of angina following a positive symptom-	/ + not mentioned

								behavioural risks for further heart disease were introduced.	infarction and revascularization) or as carers of people with heart disease. They were recruited via advert in the local press, and were employed by the local NHS Primary Care Trust.	additional homework. They were managed by a community cardiac rehabilitation nurse; had regular group and individual supervision and were able to contact the nurse for advice at any time.				limited exercise treadmill test in RACPC; does not have any exclusion criteria.	
[55]	Netherwood	2007	Pilot project	UK	Health Trainers	access to care/ Reducing health inequalities	Engage with individuals in local communities which have identified health inequalities<br>Communicate with individuals about health and health improvement<br>Enable individuals to change their behaviour to improve their health<br>Manage and organize their time and activities to support individuals in the community	specific activities for each role have been identified, but are not included in this article	Not mentioned	Not mentioned	Not mentioned	This paper uses Whitehead's framework to examine the decision to implement health trainers as a stated aim of tackling health inequalities.	Whether the creation of this new tier of workforce in the community (UKPHA, 2004) is the best way of supporting health and reducing health inequalities remains to be seen.	These areas also tend to have higher than average levels of unemployment, more single parent families and a higher	The implementation of Health Trainers appears to tackle health from an individual behavioural perspective. + not mentioned

														proportion of black and minority ethnic groups, especially Pakistani, Bangladeshi and Caribbean communities	
[56]	Thompson et al.	2018	Pilot study for Randomized controlled trial	UK	Health Trainers	provide support for lifestyle change, enhance mental well-being and signpost to appropriate services	HTs' main role is to provide one-to-one support to people in disadvantaged areas to facilitate health behaviour change. The HT role has been adapted for specific populations, including offenders and smokers, with early signs that the support is acceptable and feasible	Provide one-on-one sessions with the participant, provide support, help participants understand the inter-relationship between health behaviours such as smoking, alcohol use, diet, physical activity and their relationship to mental well-being, help built positive behaviours	Not mentioned	A training package was delivered to the HTs on the project focusing on the core competencies of an HT as outlined in the HT handbook9 with training in the 5WWB. HTs are trained to help participants understa	Not mentioned	This study aims to determine the feasibility and acceptability of conducting a randomised trial and delivering a health trainer intervention to people receiving community supervision in the UK.	/	People with experience of the criminal justice system. If they have served a custodial sentence, then they have to have been released for at least 2 months. The	the scope of HTs has been extended to prison and probation settings with promising findings, 11 especially when the HT has personal experience of the CJS + not

										nd the inter-relationship between health behaviours such as smoking, alcohol use, diet, physical activity and their relationship to mental well-being and other positive and negative behaviours, including substance use.				supervision period must have at least 7 months left at recruitment. Participants are interested in receiving support to change diet, physical activity, alcohol use and smoking and/or improve mental well-being. The primary outcome is mental well-being with second	mentioned

														ary outcomes related to smoking, physical activity, alcohol consumption and diet.	
