# Supplementary material for: The role of community health workers in primary healthcare in the WHO-EU region: a scoping review
Source: Int J Equity Health. 2023 Jul 20;22:134. doi: 10.1186/s12939-023-01944-0 (PMC10357780; doi:10.1186/s12939-023-01944-0)
Supplement: Supplementary file 3 — Additional File 3: ICROMS sheet & scoring system [file 12939_2023_1944_MOESM3_ESM.pdf]

ICROMS Tool									
	Quality criteria		Study design						
	Dimension	Specific criteria	RCT/cRCT	CBA	CITS	NCITS	NCBA	CS	QUAL
1	Clear aims and justification	A. Clear statement of the aims of the research?	✓✓	✓✓	✓✓	✓✓	✓✓	✓✓	✓✓
		B. Rationale for number of pre- and post-intervention points or adequate baseline measurement	✗	✗	✓	✓✓	✓✓	✗	✗
		C. Explanation for lack of control group	✗	✗	✗	✓	✓	✗	✗
		D. Appropriateness of qualitative methodology	✗	✗	✗	✗	✗	✗	✓
		E. Appropriate study design	✗	✗	✗	✗	✗	✗	✓✓
2	Managing bias in sampling or between groups	A. Sequence Generation	✓✓	✗	✗	✗	✗	✗	✗
		B. Allocation Concealment	✓✓	✗	✗	✗	✗	✗	✗
		C. Justification for sample choice	✗	✗	✗	✓✓	✓✓	✗	✗
		D. Intervention and control group selection designed to protect against systematic difference/selection bias	✗	✓✓	✗	✗	✗	✗	✗
		E. Comparability of groups	✗	✗	✗	✗	✗	✓✓	✗
		F. Sampling and recruitment	✗	✗	✗	✗	✗	✗	✓✓
3	Managing bias in outcome measurements and Blinding	A. Blinding	✓✓	✗	✗	✗	✗	✗	✗
		B. Baseline measurement – protection against selection bias	✗	✓✓	✗	✗	✗	✗	✗
		C. Protection against contamination	✗	✓✓	✗	✗	✗	✗	✗
		D. Protection against secular changes	✗	✗	✓✓	✗	✗	✗	✗
		E. Protection against detection bias: Blinded assessment of primary outcome measures	✓	✓	✓	✓	✓	✓	✗
		F. Reliable primary outcome measures	✓	✓	✓	✓	✓	✓	✓
		G. Comparability of outcomes	✗	✗	✗	✗	✗	✓✓	✗
4	Managing bias in follow-up	A. Follow-up of subjects (protection against exclusion bias)	✓	✗	✗	✗	✗	✗	✗
		B. Follow-up of patients or episodes of care	✓	✗	✗	✗	✗	✗	✗
		C. Incomplete outcome data addressed	✓	✓	✓	✓	✓	✓✓	✓
5	Managing bias in other study aspects	A. Protection against detection bias: Intervention unlikely to affect data collection	✓	✓	✓	✓	✓	✗	✗
		B. Protection against information bias	✗	✗	✗	✗	✗	✓	✗
		C. Data collection appropriate to address research aims	✗	✗	✗	✗	✗	✗	✓
		D. Attempts to mitigate effects of no control	✗	✗	✗	✓✓	✓✓	✗	✗
6	Analytical rigour	A. Sufficient data points to enable reliable statistical inference	✗	✗	✓✓	✗	✗	✗	✗
		B. Shaping of intervention effect specified	✗	✗	✓	✗	✗	✗	✗

		C. Analysis sufficiently rigorous/free from bias	✓	✓	✓	✓	✓	✓	✓
7	Managing bias in reporting/ethical considerations	A. Free of selective outcome reporting	✓	✓	✓	✓	✓	✓	✓
		B. Limitations addressed	✓	✓	✓	✓	✓	✓	✓
		C. Conclusions clear and justified	✓	✓	✓	✓	✓	✓	✓
		D. Free of other bias	✓	✓	✓	✓	✓	✓	✓
		E. Ethics issues addressed	✓	✓	✓	✓	✓	✓	✓
Total score/ maximum score									
Overall appraisal									

Applicability of quality criteria to each study design: ✓ = Criteria to be included in quality assessment for study design; ✓✓ Mandatory criteria to be met quality assessment; ✗ = Criteria not to be applied in quality assessment for study design. Study designs: RCT = randomised controlled trial; CBA = controlled before-after; CITS = controlled interrupted time series; CS = cohort study; NCITS = non-controlled interrupted time series; NCBA = non-controlled before-after; QUAL = qualitative.

**ICROMS Scoring System**			
Study design	Minimum score	Maximum score	Scoring system
RCT, cRCT	22	32	Scores applicable to each criteria: Yes (criterion met) = 2 points; Unclear (unclear whether or not the criterion is met) = 1 point; No (criterion not met) = 0 points .
CBA	18	28	
CITS	18	30	
NCITS	22	30	
NCBA	22	30	
CS (cohort)	18	26	
QUAL	16	26	
Other designs	Max score - 10	Number of applicable criteria x2	

Study designs: RCT = randomised controlled trial; CBA = controlled before-after; CITS = controlled interrupted time series; CS = cohort study; NCITS = non-controlled interrupted time series; NCBA = non-controlled before-after; QUAL = qualitative.

ICROMS tool applied to the included studies

Authors	Study design	1A	1B	1C	1D	1 E	2A	2B	2C	2D	2E	2 F	3A	3B	3C	3D	3 E	3 F	3G	4A	4B	4C	5A	5B	5C	5D	6A	6B	6C	7A	7B	7C	7D	7E	Score	Appraisal
Allen-Collinson et al.	QUAL	Yes	N.A.	N.A.	Yes	Yes	N.A.	N.A.	N.A.	N.A.	N.A.	No	N.A.	N.A.	N.A.	N.A.	N.A.	Yes	N.A.	N.A.	N.A.	Unclear	N.A.	N.A.	Unclear	N.A.	N.A.	N.A.	Yes	Unclear	No	Yes	Unclear	Yes	18/26	Moderate
Ball & Nasr	QUAL	Yes	N.A.	N.A.	Yes	Yes	N.A.	N.A.	N.A.	N.A.	N.A.	Unclear	N.A.	N.A.	N.A.	N.A.	N.A.	Yes	N.A.	N.A.	N.A.	Unclear	N.A.	N.A.	Unclear	N.A.	N.A.	N.A.	Unclear	Unclear	No	Yes	Unclear	Yes	18/26	Moderate
Begh et al. (1)	cRCT	Yes	N.A.	N.A.	N.A.	N.A.	Yes	Yes	N.A.	N.A.	N.A.	N.A.	Unclear	N.A.	N.A.	N.A.	Yes	No	N.A.	Yes	Yes	Unclear	Yes	N.A.	N.A.	N.A.	N.A.	N.A.	Yes	Yes	No	Yes	Unclear	Yes	25/32	Moderate
Begh et al. (2)	QUAL	Yes	N.A.	N.A.	Yes	Yes	N.A.	N.A.	N.A.	N.A.	N.A.	Yes	N.A.	N.A.	N.A.	N.A.	N.A.	Yes	N.A.	N.A.	N.A.	No	N.A.	N.A.	Yes	N.A.	N.A.	N.A.	Yes	Unclear	Yes	Yes	No	Yes	21/26	High
Brady & Keogh	QUAL	Yes	N.A.	N.A.	Yes	Yes	N.A.	N.A.	N.A.	N.A.	N.A.	Unclear	N.A.	N.A.	N.A.	N.A.	N.A.	Unclear	N.A.	N.A.	N.A.	No	N.A.	N.A.	Yes	N.A.	N.A.	N.A.	Unclear	Unclear	Yes	Yes	Unclear	Yes	19/26	Moderate
Brown et al.	QUAL	Yes	N.A.	N.A.	Yes	No	N.A.	N.A.	N.A.	N.A.	N.A.	Unclear	N.A.	N.A.	N.A.	N.A.	N.A.	No	N.A.	N.A.	N.A.	Yes	N.A.	N.A.	Unclear	N.A.	N.A.	N.A.	Unclear	Unclear	Yes	Yes	Unclear	No	15/26	Low
Carver et al.	QUAL	Yes	N.A.	N.A.	Yes	Unclear	N.A.	N.A.	N.A.	N.A.	N.A.	Unclear	N.A.	N.A.	N.A.	N.A.	N.A.	Unclear	N.A.	N.A.	N.A.	No	N.A.	N.A.	Unclear	N.A.	N.A.	N.A.	Unclear	Unclear	Yes	Yes	Unclear	No	16/26	Moderate
Cook & Wills	QUAL	Yes	N.A.	N.A.	Yes	Yes	N.A.	N.A.	N.A.	N.A.	N.A.	Unclear	N.A.	N.A.	N.A.	N.A.	N.A.	Yes	N.A.	N.A.	N.A.	Unclear	N.A.	N.A.	Yes	N.A.	N.A.	N.A.	Yes	Unclear	No	Yes	Unclear	Yes	20/26	High
Furze et al.	RCT	Yes	N.A.	N.A.	N.A.	N.A.	Yes	No	N.A.	N.A.	N.A.	N.A.	Yes	N.A.	N.A.	N.A.	Unclear	No	N.A.	Yes	Yes	Yes	Yes	N.A.	N.A.	N.A.	N.A.	N.A.	Yes	Yes	Yes	Yes	No	Yes	25/32	Moderate
Gale & Sidhu	QUAL	Yes	N.A.	N.A.	Yes	Yes	N.A.	N.A.	N.A.	N.A.	N.A.	Yes	N.A.	N.A.	N.A.	N.A.	N.A.	Yes	N.A.	N.A.	N.A.	Unclear	N.A.	N.A.	Yes	N.A.	N.A.	N.A.	Yes	Unclear	Yes	Yes	Unclear	Yes	23/26	High

Gale et al.	QUAL	Yes	N.A.	N.A.	Unclear	Yes	N.A.	N.A.	N.A.	N.A.	N.A.	Unclear	N.A.	N.A.	N.A.	N.A.	N.A.	Unclear	N.A.	N.A.	N.A.	No	N.A.	N.A.	Unclear	N.A.	N.A.	N.A.	Yes	Unclear	Yes	Yes	Unclear	Yes	18/26	Moderate
Gilworth et al.	QUAL	Yes	N.A.	N.A.	Yes	Yes	N.A.	N.A.	N.A.	N.A.	N.A.	Unclear	N.A.	N.A.	N.A.	N.A.	N.A.	Unclear	N.A.	N.A.	N.A.	No	N.A.	N.A.	Unclear	N.A.	N.A.	N.A.	Unclear	Unclear	Yes	Yes	Unclear	Yes	18/26	Moderate
Goelen et al.	RCT	Yes	N.A.	N.A.	N.A.	N.A.	Unclear	Unclear	N.A.	N.A.	N.A.	N.A.	Yes	N.A.	N.A.	N.A.	Unclear	Yes	N.A.	Unclear	No	No	Unclear	N.A.	N.A.	N.A.	N.A.	N.A.	Unclear	Unclear	Unclear	Yes	Unclear	No	15/32	Low
Hesselink & Harting	Mixed mothods	Yes	N.A.	N.A.	Yes	Yes	N.A.	N.A.	N.A.	N.A.	N.A.	Unclear	N.A.	N.A.	N.A.	N.A.	N.A.	Yes	N.A.	N.A.	N.A.	Unclear	No	N.A.	Yes	N.A.	N.A.	N.A.	Unclear	Unclear	Yes	Yes	Unclear	Yes	21/28	Moderate
Hodgins et al.	quasi-experimental	Yes	N.A.	N.A.	N.A.	Yes	N.A.	N.A.	N.A.	N.A.	N.A.	Yes	N.A.	N.A.	N.A.	N.A.	N.A.	Yes	N.A.	N.A.	N.A.	Unclear	No	No	Unclear	N.A.	N.A.	N.A.	Yes	Yes	Yes	Yes	Unclear	Yes	20/28	Moderate
Hoens et al.	Realist evaluation	Yes	N.A.	N.A.	Yes	Yes	N.A.	N.A.	N.A.	N.A.	N.A.	Unclear	N.A.	N.A.	N.A.	N.A.	N.A.	Yes	N.A.	N.A.	N.A.	Unclear	No	N.A.	Yes	N.A.	N.A.	N.A.	Yes	Unclear	Yes	Yes	No	Yes	19/28	Moderate
Kennedy et al.	QUAL	Yes	N.A.	N.A.	Yes	No	N.A.	N.A.	N.A.	N.A.	N.A.	Yes	N.A.	N.A.	N.A.	N.A.	N.A.	Unclear	N.A.	N.A.	N.A.	Unclear	N.A.	N.A.	Yes	N.A.	N.A.	N.A.	Unclear	Unclear	Yes	Yes	No	No	16/26	Moderate
Kennedy, L..	QUAL	Yes	N.A.	N.A.	Yes	Yes	N.A.	N.A.	N.A.	N.A.	N.A.	Yes	N.A.	N.A.	N.A.	N.A.	N.A.	Yes	N.A.	N.A.	N.A.	Unclear	N.A.	N.A.	Yes	N.A.	N.A.	N.A.	Yes	Unclear	No	Yes	Unclear	Yes	21/26	High
Kenyon et al.	RCT	Yes	N.A.	N.A.	N.A.	N.A.	Yes	Unclear	N.A.	N.A.	N.A.	N.A.	No	N.A.	N.A.	N.A.	Yes	Yes	N.A.	Yes	Yes	No	Unclear	N.A.	N.A.	N.A.	N.A.	N.A.	Unclear	Yes	Yes	Yes	Unclear	Yes	24/32	Moderate
Kosa et al.	Quantitative analysis	Unclear	N.A.	N.A.	N.A.	Yes	N.A.	N.A.	N.A.	N.A.	N.A.	Unclear	N.A.	No	N.A.	N.A.	N.A.	Yes	N.A.	N.A.	N.A.	Unclear	Unclear	N.A.	Yes	N.A.	N.A.	N.A.	Yes	Unclear	Yes	Yes	No	No	17/28	Low
Lopez-Sanchez et al.	Quantitative analysis	Yes	N.A.	N.A.	N.A.	Yes	N.A.	N.A.	N.A.	N.A.	N.A.	Unclear	N.A.	N.A.	N.A.	N.A.	N.A.	Yes	N.A.	N.A.	N.A.	Unclear	Unclear	N.A.	Yes	N.A.	N.A.	N.A.	Yes	Unclear	Yes	Yes	Unclear	Yes	21/26	High

Lorente et al.	QUAL	Yes	N.A.	N.A.	Yes	Yes	N.A.	N.A.	N.A.	N.A.	N.A.	Yes	N.A.	N.A.	N.A.	N.A.	N.A.	Yes	N.A.	N.A.	N.A.	Unclear	N.A.	N.A.	Yes	N.A.	N.A.	N.A.	Yes	Unclear	Yes	Yes	Unclear	Yes	23/26	High
Martró et al.	Cross sectional	Yes	N.A.	N.A.	Yes	Yes	N.A.	N.A.	N.A.	N.A.	N.A.	Unclear	N.A.	N.A.	N.A.	N.A.	N.A.	Yes	N.A.	N.A.	N.A.	Unclear	N.A.	N.A.	Unclear	N.A.	N.A.	N.A.	Unclear	Unclear	Yes	Yes	Unclear	Yes	20/26	Moderate
McWilliams et al.	QUAL	Yes	N.A.	N.A.	Yes	Yes	N.A.	N.A.	N.A.	N.A.	N.A.	Yes	N.A.	N.A.	N.A.	N.A.	N.A.	Yes	N.A.	N.A.	N.A.	Unclear	N.A.	N.A.	Yes	N.A.	N.A.	N.A.	Yes	Unclear	Yes	Yes	Unclear	Yes	23/26	High
Netherwood	commenntary	Yes	N.A.	N.A.	Unclear	Unclear	N.A.	N.A.	N.A.	N.A.	N.A.	N.A.	N.A.	N.A.	N.A.	N.A.	N.A.	Unclear	N.A.	N.A.	N.A.	N.A.	N.A.	N.A.	Unclear	N.A.	N.A.	N.A.	Unclear	Unclear	No	Unclear	Unclear	No	10/22	Low
Rämgard & Avery	QUAL	Yes	N.A.	N.A.	Yes	Yes	N.A.	N.A.	N.A.	N.A.	N.A.	Unclear	N.A.	N.A.	N.A.	N.A.	N.A.	Unclear	N.A.	N.A.	N.A.	No	N.A.	N.A.	Unclear	N.A.	N.A.	N.A.	Unclear	Unclear	Unclear	Yes	Unclear	Yes	17/26	Moderate
Roberts et al.	Costing study	Yes	N.A.	N.A.	N.A.	Yes	N.A.	N.A.	N.A.	N.A.	N.A.	Unclear	N.A.	N.A.	N.A.	N.A.	N.A.	Yes	N.A.	N.A.	N.A.	Unclear	Yes	N.A.	Yes	N.A.	N.A.	N.A.	Yes	Unclear	No	Yes	Unclear	Yes	20/26	High
South et al.	QUAL	Yes	N.A.	N.A.	Yes	Yes	N.A.	N.A.	N.A.	N.A.	N.A.	Yes	N.A.	N.A.	N.A.	N.A.	N.A.	Yes	N.A.	N.A.	N.A.	Unclear	N.A.	N.A.	Yes	N.A.	N.A.	N.A.	Yes	Unclear	Yes	Yes	Unclear	Yes	23/26	High
Stone et al.	QUAL	Yes	N.A.	N.A.	Yes	Yes	N.A.	N.A.	N.A.	N.A.	N.A.	Unclear	N.A.	N.A.	N.A.	N.A.	N.A.	Yes	N.A.	N.A.	N.A.	No	N.A.	N.A.	Yes	N.A.	N.A.	N.A.	Yes	Unclear	Yes	Yes	Unclear	Yes	19/26	Moderate
Thompson et al.	pilot for RCT	Yes	N.A.	N.A.	Yes	Yes	N.A.	N.A.	N.A.	N.A.	N.A.	Yes	N.A.	N.A.	N.A.	N.A.	N.A.	Yes	N.A.	N.A.	N.A.	Unclear	N.A.	N.A.	Unclear	N.A.	N.A.	N.A.	Yes	Unclear	Yes	Yes	Unclear	Yes	22/26	High
Vanden Bossche et al. (1)	RCT	Yes	N.A.	N.A.	N.A.	N.A.	Yes	No	N.A.	N.A.	N.A.	N.A.	Yes	N.A.	N.A.	N.A.	Unclear	No	N.A.	Yes	Yes	Yes	Yes	N.A.	N.A.	N.A.	N.A.	N.A.	Yes	Yes	Yes	Yes	No	Yes	25/32	Moderate
Vanden Bossche et al. (2)	Realist evaluation	Yes	N.A.	N.A.	Yes	Yes	N.A.	N.A.	N.A.	N.A.	N.A.	Yes	N.A.	N.A.	N.A.	N.A.	N.A.	Yes	N.A.	N.A.	N.A.	Unclear	Yes	N.A.	Yes	N.A.	N.A.	N.A.	Yes	Unclear	Yes	Yes	Unclear	Yes	25/28	High

Verhagen et al.	Quasi experimental study	Yes	N.A.	N.A.	N.A.	Yes	N.A.	N.A.	N.A.	N.A.	N.A.	Unclear	N.A.	N.A.	N.A.	N.A.	N.A.	Yes	N.A.	N.A.	N.A.	Unclear	No	No	Unclear	N.A.	N.A.	N.A.	Unclear	Unclear	Yes	Yes	Unclear	Yes	18/28	Moderate
Visram et al.	QUAL	Yes	N.A.	N.A.	Yes	Yes	N.A.	N.A.	N.A.	N.A.	N.A.	Yes	N.A.	N.A.	N.A.	N.A.	N.A.	Yes	N.A.	N.A.	N.A.	Unclear	N.A.	N.A.	Unclear	N.A.	N.A.	N.A.	Yes	Unclear	Yes	Yes	Yes	Yes	24/26	High
White et al. (1)	Mixed methods	Yes	N.A.	N.A.	Yes	Yes	N.A.	N.A.	N.A.	N.A.	N.A.	Unclear	N.A.	N.A.	N.A.	N.A.	N.A.	Unclear	N.A.	N.A.	N.A.	Unclear	No	N.A.	Yes	N.A.	N.A.	N.A.	Unclear	Unclear	Yes	Yes	Unclear	Yes	20/28	Moderate
White et al. (2)	Pilot project	Yes	N.A.	N.A.	Yes	Yes	N.A.	N.A.	N.A.	N.A.	N.A.	Yes	N.A.	N.A.	N.A.	N.A.	N.A.	Yes	N.A.	N.A.	N.A.	Unclear	N.A.	N.A.	Unclear	N.A.	N.A.	N.A.	Yes	Unclear	Yes	Yes	Unclear	Yes	22/26	High
Wildman & Wildman	CS	Yes	N.A.	N.A.	N.A.	N.A.	N.A.	N.A.	N.A.	N.A.	Yes	N.A.	N.A.	N.A.	N.A.	N.A.	No	Yes	Yes	N.A.	N.A.	No	N.A.	Unclear	N.A.	N.A.	N.A.	N.A.	Yes	Yes	Yes	Yes	Unclear	Yes	20/26	Moderate
Wrede et al.	CS	Yes	N.A.	N.A.	N.A.	N.A.	N.A.	N.A.	N.A.	N.A.	No	N.A.	N.A.	N.A.	N.A.	N.A.	No	No	Yes	N.A.	N.A.	Unclear	N.A.	Unclear	N.A.	N.A.	N.A.	N.A.	Yes	Yes	Yes	Yes	Yes	Yes	18/26	Moderate
Yoeli & Catan	QUAL	Yes	N.A.	N.A.	Yes	Yes	N.A.	N.A.	N.A.	N.A.	N.A.	Unclear	N.A.	N.A.	N.A.	N.A.	N.A.	Unclear	N.A.	N.A.	N.A.	No	N.A.	N.A.	Yes	N.A.	N.A.	N.A.	Yes	Unclear	Yes	Yes	Unclear	Yes	20/26	High
Yorick et al.	QUAL	Unclear	N.A.	N.A.	Yes	Unclear	N.A.	N.A.	N.A.	N.A.	N.A.	Unclear	N.A.	N.A.	N.A.	N.A.	N.A.	Yes	N.A.	N.A.	N.A.	Unclear	N.A.	N.A.	N.A.	N.A.	N.A.	N.A.	Yes	Yes	Yes	Yes	Unclear	No	17/26	Moderate

Study designs: (c)RCT = (cluster) randomised controlled trial; CBA = controlled before-after; CITS = controlled interrupted time series; CS = cohort study; NCITS = non-controlled interrupted time series; NCBA = non-controlled before-after; QUAL = qualitative.
